# Atlas of gross, histologic, and immunohistologic anatomy of the lymphoid system in Egyptian rousettes (*Rousettus aegyptiacus*)

**DOI:** 10.1186/s13567-026-01786-y

**Published:** 2026-08-27

**Authors:** Jessica A. Elbert, Brian R. Amman, Tara K. Sealy, Patrick Atimnedi, Jonathan S. Towner, Elizabeth W. Howerth

**Affiliations:** 1 Department of Pathology, University of Georgia College of Veterinary Medicine, Athens, GA USA; 2 Viral Special Pathogens Branch, Division of High-Consequence Pathogens and Pathology, Centers for Disease Control and Prevention, Atlanta, GA USA; 3 Uganda Wildlife Authority, Kampala, Uganda

**Keywords:** Egyptian rousette, *Rousettus aegyptiacus*, histology, immunology, lymphoid system, reservoir

## Abstract

Chiropterans exhibit remarkable immunological adaptations that facilitate their function as competent reservoir hosts for diverse high-consequence zoonotic viruses while maintaining apparent clinical health. The Egyptian rousette (*Rousettus aegyptiacus*), a frugivorous pteropodid species, represents the sole confirmed natural reservoir for Marburg and Ravn viruses (family Filoviridae), yet comprehensive characterization of its lymphoid system architecture remains absent from the literature. This investigation presents the first systematic gross, histological, and immunohistochemical atlas of lymphoid tissues in this epidemiologically critical species, encompassing primary (bone marrow, thymus) and secondary lymphoid tissues (lymph nodes, spleen, mucosa-associated lymphoid tissue). Tissues from fetal, juvenile, and adult specimens were systematically evaluated to characterize age-related variations in lymphoid architecture and cellular composition dynamics. Immunohistochemical analyses employing cross-reactive antibodies against CD3, CD79a, Iba1, and Granzyme B elucidated the spatial distribution and organizational patterns of key lymphocyte populations and antigen-presenting cells across distinct lymphoid compartments. While overall lymphoid tissue organization demonstrated conservation relative to other mammalian species, several distinctive morphological features were identified, including prominently developed splenic marginal zones, extensive gut-associated lymphoid tissue networks, and organized nasopharyngeal and bronchial lymphoid structures. These anatomical specializations likely represent species-specific evolutionary adaptations underlying viral tolerance mechanisms and immunological equilibrium maintenance during persistent viral exposure. This comprehensive atlas provides an essential foundational resource for veterinary pathologists, comparative immunologists, and infectious disease investigators conducting research with *R. aegyptiacus* bat populations. By establishing anatomically and immunologically detailed reference standards for lymphoid tissues in this critical reservoir species, this work supports interpretation of experimental infection studies and may help guide future investigations of host–pathogen interactions, reservoir competence, and zoonotic spillover dynamics at wildlife–human interfaces.

## Introduction

Bats (order Chiroptera) constitute the second most diverse mammalian order and occupy an extraordinary range of ecological niches across global ecosystems, fulfilling critical ecological functions as pollinators and seed dispersers [1]. In recent decades, chiropterans have garnered escalating scientific attention owing to their remarkable immunological adaptations and their established role as natural reservoir hosts for numerous zoonotic, frequently high-consequence pathogens, including Hendra virus, Nipah virus, severe acute respiratory syndrome coronavirus (SARS-CoV), and Marburg virus [2–7]. The increasing emergence and recognition of these pathogens has intensified research interest in understanding the unique immunological and virological mechanisms that enable bats to harbor potentially lethal viruses with minimal to asymptomatic clinical disease in themselves [3, 8–10].

The lymphoid system plays a central role in host immunity, encompassing both primary organs, bone marrow, and thymus, and secondary organs such as the spleen, lymph nodes, and mucosa-associated lymphoid tissue (MALT) [11]. In bats, these structures are broadly conserved, but their anatomic distribution, cellular composition, and developmental trajectories appear to differ from those of other mammals and can differ between individual animals of the same species. For instance, variation has been noted between a few bat species in the presence of gut-associated lymphoid tissue (GALT), cellular populations in splenic tissue, and anatomical distribution of lymph nodes [11]. Despite the critical importance of lymphoid architecture in understanding immune function and host–pathogen interactions, the structure and function of chiropteran lymphoid systems remain incompletely characterized. Most available data derive from limited species representation and focus predominantly on functional immunology rather than comprehensive tissue-level anatomical characterization.

The Egyptian rousette (*Rousettus aegyptiacus*; ERB), is a cave-roosting, frugivorous pteropod bat with extensive geographical distribution across sub-Saharan Africa and portions of the Middle East [12]. This species is epidemiologically significant as the sole confirmed natural reservoir host for the orthomarburgviruses Marburg virus and Ravn virus (MARV; RAVV; family Filoviridae; genus *Orthomarburgvirus*) [6, 7, 13]. These closely related filoviruses serve as the etiological agents of Marburg virus disease (MVD), a severe viral hemorrhagic fever characterized by human-to-human transmission and case fatality ratios reaching 90% [14, 15]. Beyond its role as an orthomarburgvirus reservoir, the ERB serves as a vertebrate reservoir for Kasokero virus (KASV; family Orthonairoviridae, genus *Orthonairovirus*) [16, 17] and is presumed to function as a natural reservoir for Sosuga virus (SOSV; family Paramyxoviridae, genus *Pararubulavirus*) [18]. This remarkable capacity to harbor multiple, frequently high-consequence pathogens while maintaining population health underscores the species’ unique immunological adaptations and reservoir competence mechanisms.

Given the central role of ERBs in filovirus ecology and zoonotic spillover dynamics, comprehensive understanding of ERB-specific lymphoid tissue architecture is essential for elucidating host–pathogen interaction mechanisms and informing evidence-based public health strategies. This investigation presents a comprehensive gross, histological, and immunohistochemical atlas of the lymphoid system in ERBs, with particular emphasis on juvenile specimens representing the established experimental model demographic. This work establishes standardized reference criteria for the anatomy and cellular architecture of primary and secondary lymphoid organs, providing essential context for interpreting histopathological findings in both natural infection scenarios and experimental settings.

This atlas supports ongoing research endeavors in comparative pathology, disease ecology, and host–pathogen interaction dynamics by providing foundational anatomical knowledge necessary for understanding immune responses in this critical reservoir species. By establishing detailed morphological and immunological baselines, this work enhances the interpretive power of experimental infection studies, facilitates accurate pathological assessment, and contributes to our fundamental understanding of the mechanisms underlying viral tolerance and reservoir competence in chiropteran species.

## Materials and methods

### Study population and specimen selection

This investigation utilized six Egyptian rousette specimens representing three developmental stages: two fetuses from free-ranging dams, two juvenile specimens, and two adult individuals from captive-born populations [19]. All capture, processing, and experimental procedures were conducted in strict accordance with institutionally approved animal care and use protocols, as previously detailed [6, 13]. Specimen collections were performed under Uganda Wildlife Authority approval and adhered to American Veterinary Medical Association euthanasia guidelines and National Research Council recommendations for laboratory animal care and use [6].

### Fetal specimens

Two near-term ERB fetuses (approximate embryonic development stages 24–25 [20]) were collected during 2008–2009 as components of Centers for Disease Control and Prevention (CDC) investigations to identify orthomarburgvirus natural reservoir hosts. Sex determination was not performed on fetal specimens. Intact fetal carcasses were fixed in 10% neutral buffered formalin at the time of collection. Necropsies were conducted in 2023, with systematic collection of representative sections from primary and secondary lymphoid tissues.

### Juvenile specimens

Two 6-month-old juvenile ERBs (one male, one female) served as negative controls in an experimental study conducted at the CDC and were euthanized at study termination [18]. Cranial and abdominal ± thoracic cavities were exposed to facilitate 10% neutral buffered formalin penetration, with carcasses preserved as intact specimens. Necropsies were performed with systematic collection of representative primary and secondary lymphoid tissue sections.

### Adult specimens

Two adult ERBs (male, 10 years; female, 12 years) were selected from the managed care research colony maintained at the CDC. Prior to euthanasia, blood samples (≤ 10 μL) were collected via cephalic vein venipuncture from the propatagium and immediately processed to prepare four to five thin blood smears per specimen on glass slides. Blood smears were inactivated through 20-min submersion in 10% neutral buffered formalin and stained using Epredia Shandon Kwik-Diff Stains (Thermo Fisher Scientific, Waltham, MA, USA). Euthanasia procedures were performed as previously described [18].

### Histological processing

Tissue specimens from all animals were processed using standard histological protocols and embedded in liquid paraffin. Serial sections were cut at 4 μm thickness, mounted on glass slides, and stained with hematoxylin and eosin (H&E) for routine morphological evaluation.

### Immunohistochemical analysis

Immunohistochemical characterization was performed on formalin-fixed, paraffin-embedded (FFPE) sections of all primary and secondary lymphoid tissues using automated staining protocols (Nemesis 3600, Biocare Medical, Concord, CA, USA) at the University of Georgia Histology Laboratory. The immunohistochemical panel was designed to characterize key immune cell populations and included ionized calcium-binding adapter molecule 1 (Iba1), a pan-macrophage and dendritic cell marker; CD3, a T cell marker; CD79a, a B cell marker; and Granzyme B, a natural killer cell and cytotoxic T lymphocyte marker. For the Iba1 protocol, a rabbit polyclonal antibody directed against Iba1 (1:8000 final dilution; Wako, Richmond, VA, USA; catalog #019–19741) was incubated on the tissue for 60 min. For the CD3 protocol, a rabbit polyclonal antibody targeting the CD3 epsilon subunit (1:1000 final dilution; Dako, Carpinteria, CA, USA; catalog #A0452) was incubated on the tissue for 60 min. For the CD79a protocol, a mouse monoclonal antibody directed against CD79a (1:50 final dilution; Biocare Medical, Pacheco, CA, USA; catalog #CM067C) was incubated on the tissue for 60 min. For the Granzyme B protocol, a rabbit polyclonal antibody directed against Granzyme B gene 3002 (1:200 final dilution; Spring BioScience/Roche, Pleasanton, CA, USA; catalog #E2582) was incubated on the tissue for 60 min.

Antigen retrieval for all immunohistochemical stains was performed using Antigen Retrieval Citra Solution 10X (Biogenex/Thermo Fisher Scientific, Waltham, MA, USA) at 1:10 dilution for 15 min at 110 °C. Biotinylated rabbit or mouse secondary antibodies (Vector Laboratories, Burlingame, CA, USA) were employed for primary antibody detection. Immunoreactions were visualized using 3,3'-diaminobenzidine substrate (DAB; Dako, Santa Clara, CA, USA) with 12-min development time. Sections were counterstained with hematoxylin and coverslipped using standard protocols.

Additional B cell markers including CD20, CD21, and Pax-5 were attempted but failed to demonstrate successful cross-reactivity with ERB tissues under the conditions tested. Positive control tissues are listed in Table 1.

**Table 1:** **Compiled information on antibodies used on Egyptian rousette (*Rousettus aegyptiacus*) tissues**

Antibody	Host	Source, catalog ID	Antigen retrieval	Dilution	Chromogen	Positive control	Successful	Ref.
Anti-cholineacetyl transferase	R	Chemicon, AB143	NS	1:1500	DAB	NS	Yes	[77]
Anti-serotonin	R	Chemicon, AB938	NS	1:7500	DAB	NS	Yes	[77]
Anti-tyrosine hydroxylase (TH)	R	Chemicon, AB151	NS	1:6000	DAB	NS	Yes	[77]
Caspase 3	R	Biocare Medical, CP229B	High pH antigen retrieval 15 min at 110 °C	1:8000	DAB	R lymph node	Yes	[63]
CD3	R	Dako, A0452	Citrate 15 min at 115 °C; NS	1:1000 or NS	DAB or NS	C lymph node	Yes, No [78]	[63, 78], Current study
CD20	R	Biocare Medical, ACR3004B	Citrate 15 min at 115 °C	1:500	DAB	C lymph node	No	Current study
CD21	M	Cell Marque, 12R-16	Citrate 15 min at 115 °C	1:75	DAB	C lymph node	No	Current study
CD79a	M	Dako, M7051; Biocare Medical, CM067C	Reveal Decloaker 15 min at 115 °C; NS	1:50 or NS	DAB	C lymph node	Yes, No [78]	[78], Current study
CD117 (c-kit)	R	Dako, A4502	Citrate buffer	1:200	DAB	C	Yes	[57]
Cytokeratin (Pan)	M, NS	Dako; NS	Citrate buffer; NS	1:100 or NS	NovaRed or NS	C haired skin; C; sebaceous epithelial cells; NS	Yes, No [59]	[58, 59, 61, 78]
Cytokeratin 19	M	Dako, IR615	NS	Ready to use	DAB	C, ERB liver	Yes	[60]
Desmin	R, M	Dako, M0760; Dako, Clone D33; NS	Citrate buffer; no treatment; NS	1:200 or NS	DAB, NovaRed, or NS	Equine cardiomyocytes; vascular and intestinal smooth muscle; C	Yes	[57–59, 78]
E-cadherin	M	BD Biosciences, 610,181	Citrate 15 min at 115 °C	1:500	DAB	M skin	Yes	Unpublished data
Ebola virus	R	Viral Special Pathogens Branch, CDC	Proteinase K 15 min at RT, followed by Ultra V Block 10 min at RT	1:250	Fast Red	NS	Yes	[64]
Factor VIIIRA	R	Cell Marque, 250A-18	Citrate 15 min at 110 °C	Ready to use	DAB	R lymph node	Yes	Unpublished data
Glypican-3	M	Santa Cruz Biotechnology, SC-65443	NS	1:50	DAB	C, ERB liver	Yes	[60]
Granzyme B	R	Spring BioScience, E2582	Citrate 15 min at 110 °C	1:200	DAB	Feline intestinal T-cell lymphoma	Yes	Current study
HepPar-1	M	Dako, IR624	NS	Ready to use	DAB	C, ERB liver	Yes	[60]
Human papillomaviruses (HPV) 1, 6, 11, 16, 18, 31	M	Chemicon	NS	NS	NS	NS	Yes	[62]
Iba1	R	WAKO, 019–19741	Citrate 15 min at 115 °C; NS	1:1000, 1:8000	DAB	C brain; NS	Yes	[60, 63], Current study
Ki-67	M	Dako, IR626	NS	Ready to use	DAB	C, ERB liver	Yes	[60]
Laminin	R	Dako, Z0097	Proteinase K	1:100	DAB	C	Yes	[57]
Marburg virus	R	Viral Special Pathogens Branch, CDC	Proteinase K 15 min at RT, followed by Ultra V Block 10 min at RT	1:250	Fast Red	NS	Yes	[7, 64]
Neuronal nuclear antigen (NeuN)	GP	MilliporeSigma, ABN90P	NS	1:2000	DAB or Streptavidin	NS	Yes	[79]
Pax-5	M	Santa Cruz Biotechnology, SC-13146	Citrate 15 min at 115 °C	1:1500	DAB	C spleen	No	Current study
S100	R	Dako, Z0311	Citrate buffer	1:100 or 1:800	DAB or NovaRed	C	Yes	[57, 58]
Smooth muscle actin (SMA)	M	Dako, M0851	Citrate 15 min at 115 °C, or no treatment, or NS	1:100, 1:200, or 1:1500	DAB, NovaRed, or NS	M intestine; equine cardiomyocytes; C; NS	Yes	[57–59], Unpublished data
Sosuga virus	R	Genscript	Reveal Decloaker 15 min at 115 °C	1:2000	Fast Red	SOSV-infected Vero cell pellet	Yes	[63]
Vimentin	M	Dako, M0720; Dako Clone V9; Dako, Clone 3B4; NS	Proteinase K, or Citrate buffer, or NS	1:100, 1:200	DAB, NovaRed, or NS	C haired skin; canine; vascular and intestinal smooth muscle	Yes, No [58]	[57–59, 78]
*Yersinia pseudotuberculosis* serotypes O1, O2, O3, O4, O5 and O6	R	Denka-Seiken Co	NS	NS	NS	NS	Yes	[65]
*Yersinia enterocolitica* serotypes O1–2, O3, O5, O8 and O9	R	Denka-Seiken Co	NS	NS	NS	NS	Yes	[65]

Information provided includes antibodies utilized in this study, published in the general literature, and used for unpublished diagnostic evaluations.

*NS* not stated, *RT* room temperature, *R* rabbit, *M* mouse, *C* canine, *GP* guinea pig.

### Lymphoid anatomy of the Egyptian rousette

Previous gross and histological investigations have demonstrated that chiropteran species have immune cellular populations and primary and secondary lymphoid organ architectures fundamentally similar to those observed in other mammalian orders [21–25]. Primary lymphoid organs, encompassing bone marrow and thymus, serve as anatomical sites where lymphocytes initially express antigen receptors and achieve phenotypic and functional maturation [26]. Secondary lymphoid organs, including lymph nodes, spleen, and mucosa-associated lymphoid tissue (MALT) components, function as specialized microenvironments where lymphocyte responses to foreign antigens are initiated and undergo clonal expansion [26]. The subsequent sections provide detailed characterization of the structure and function of each primary and secondary lymphoid organ, emphasizing relevant findings from ERB-specific investigations and comparative mammalian studies.

### Bone marrow

Evaluation of hematopoietic tissues presents unique challenges in smaller mammalian species. While definitive bone marrow characterization traditionally requires cytological aspirates or smears, systematic histological evaluation of bone marrow sections provides critical information regarding tissue architecture, cellular composition, and hematopoietic functional status. Although ERBs are unlikely to serve as standard models for hematological disorder investigations, comprehensive understanding of bone marrow architecture remains essential for thorough post-mortem analyses and complete lymphoid system characterization.

When integrated with complete blood cell count (CBC) data, histological bone marrow examination provides crucial hematopoietic system information that may be undetectable through peripheral blood evaluation alone. In ERBs, limited blood volume constraints preclude serial blood chemistry assessments.

Bone marrow, a primary lymphoid organ, occupies central cavities within axial and long bones, comprising hematopoietic tissue islands and adipocytes distributed within trabecular bone frameworks [26, 27]. As the principal post-fetal hematopoietic organ, bone marrow serves as the origination site for diverse cellular lineages including erythrocytes, granulocytes, monocytes, dendritic cells, mast cells, platelets, B and T lymphocytes, and innate lymphoid cells, all derived from common hematopoietic stem cell populations [26]. The endosteal lining comprises reticular connective tissue interspersed with osteoblasts and osteoclasts [27]. Vascular supply to long bones occurs through nutrient canals, whereby nutrient arteries enter marrow cavities, subsequently branching into arteriolar and capillary networks that anastomose with venous sinuses before draining through nutrient veins [27].

Hematopoiesis represents a highly compartmentalized process characterized by distinct anatomical organization. Erythropoiesis occurs within well-defined erythroblastic islands, granulopoiesis develops in less distinct but adjacent cellular foci, and megakaryopoiesis localizes adjacent to sinusoidal endothelium [27]. Hematopoietic cell production is regulated by pluripotent stem cells differentiating into myeloid or lymphoid lineages, with subsequent maturation processes influenced by lineage-specific growth factors and cytokine signaling cascades [26, 27].

**Figure 1:**
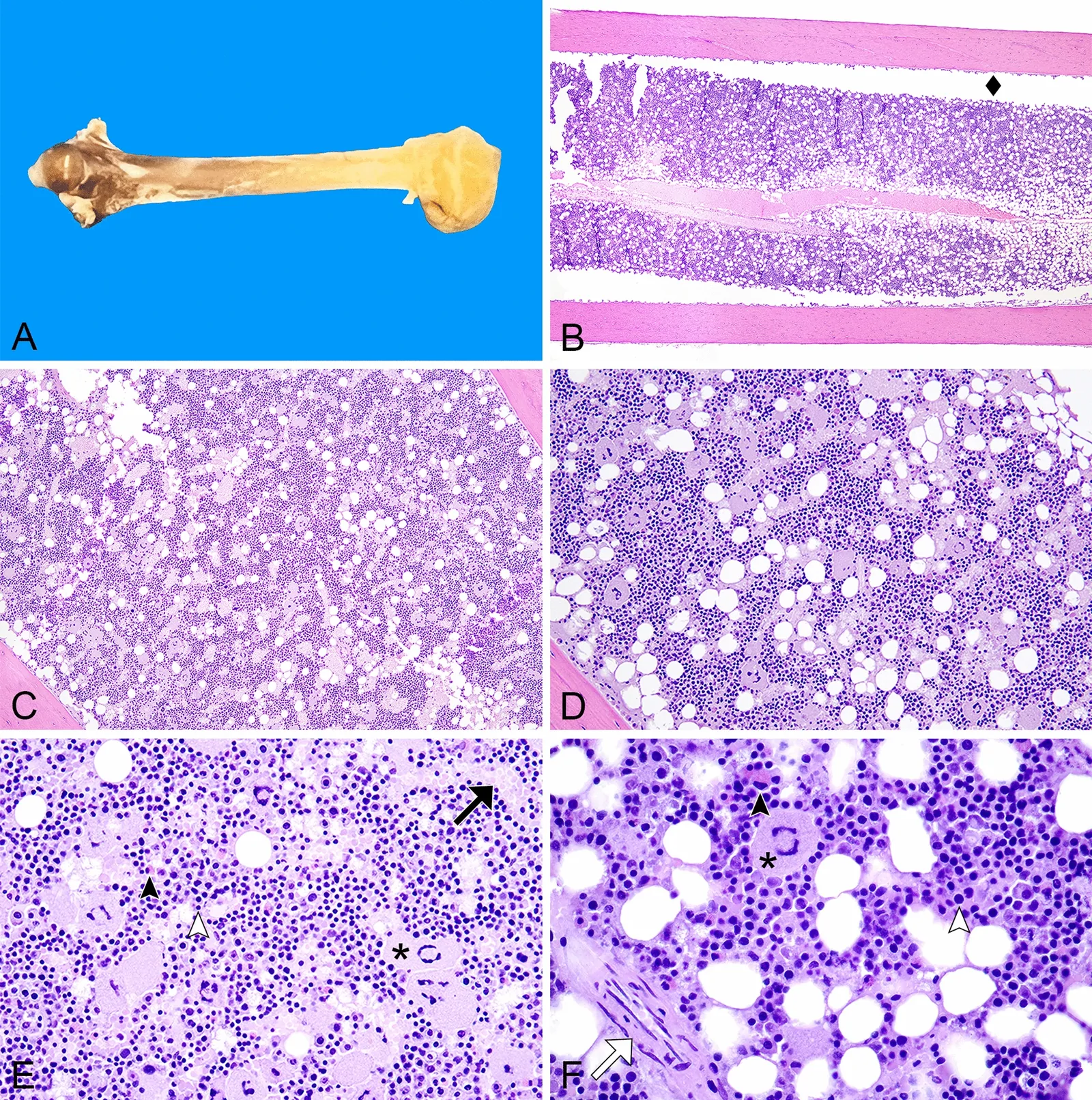
**Gross and histologic features of hematopoietic bone marrow in a 6-month-old Egyptian rousette (*Rousettus aegyptiacus*)**. **A** Gross image of a femur, with red-brown hematopoietic bone marrow extending from the femoral head (left) to mid-diaphysis. **B** Longitudinal section of femoral bone marrow extending from the proximal (left) to mid-diaphysis, showing a large central artery and vein and increased presence of adipocytes toward the mid-diaphysis. Shrinkage artifact (diamond) of bone marrow away from cortical bone is an artifact associated with histologic processing of the bone. 2×, hematoxylin and eosin (HE). **C**, **D** Representative example of bone marrow cellularity in a juvenile ERB. Bone marrow contains clusters of hematopoietic cells admixed with adipocytes. **C** 10×, HE. **D** 20×, HE. **E** The highly cellular bone marrow has areas of myeloid (black arrowhead) and erythroid (white arrowhead) hematopoiesis as well as megakaryocytes (asterisk) and a venous sinus (black arrow). Degeneration of the megakaryocyte nuclei is reflective of artifactual change from fixation procedures (i.e., fixing the limb whole versus opening the marrow cavity to be directly exposed to 10% neutral buffered formalin). 40×, HE. **F** Higher magnification image showing myeloid (black arrowhead) and erythroid (white arrowhead) hematopoiesis with admixed adipocytes, as well as megakaryocytes (asterisk) and a nutrient artery (white arrow). 60×, HE.

Comprehensive hematopoietic evaluation integrates multiple analytical approaches including peripheral blood analysis (CBC and differential counts), bone marrow cytological smears, and systematic histopathological assessment. Bone marrow histological evaluation provides essential information regarding tissue architecture, overall cellularity, myeloid:erythroid ratio estimation, cellular lineage identification, iron storage assessment, and detection of pathological alterations including neoplastic processes or inflammatory changes [27]. In canines, bone marrow biopsies are typically obtained from iliac crest, sternal, proximal humeral, or femoral sites, while in rodents, active hematopoietic sites include sternal, costal, humeral, and proximal femoral locations (Figure 1A) [27]. Normal marrow composition demonstrates age- and site-specific variation, with adipose tissue and hematopoietic tissue proportions varying accordingly.

In formalin-fixed, paraffin-embedded, and decalcified HE-stained tissue sections, mature erythroid and myeloid cells, adipocytes, mast cells, and megakaryocytes are readily identifiable, although hematopoietic stem cells and immature precursor populations are not consistently distinguishable (Figure 1B–F). Erythroid cellular populations exhibit characteristically dense, basophilic nuclei with progressive cytoplasmic eosinophilia during maturation processes. Granulocytic cells demonstrate large, bean-shaped, vesicular nuclei that are less basophilic compared with erythropoietic cell populations [27]. Megakaryocytes are distinguished by their substantial cellular size and characteristic multilobulated nuclear morphology [27]. Comprehensive discussion of fixation protocols, decalcification techniques, and tissue processing and staining methodologies is available in established references [27].

Histopathological changes documented in bone marrow of laboratory animal models include diverse alterations including: increased nucleated cell populations in response to elevated cellular demand; hematopoietic cell depletion, hypocellularity, hypoplasia, or atrophy; hematopoietic cellular dysplasia; focal stromal cell hyperplasia and myelostromal proliferation; focal lipomatosis and fibrotic changes; fibro-osseous lesions and fibrous osteodystrophy; tissue necrosis and degenerative processes; inflammatory alterations involving erythrophagocytosis alongside lymphocytic and plasma cell infiltration; primary neoplastic processes affecting hematopoietic, stromal, or endothelial cell populations; and secondary neoplastic involvement from distant metastatic disease or locally invasive tumor processes [28].

### Thymus

**Figure 2:**
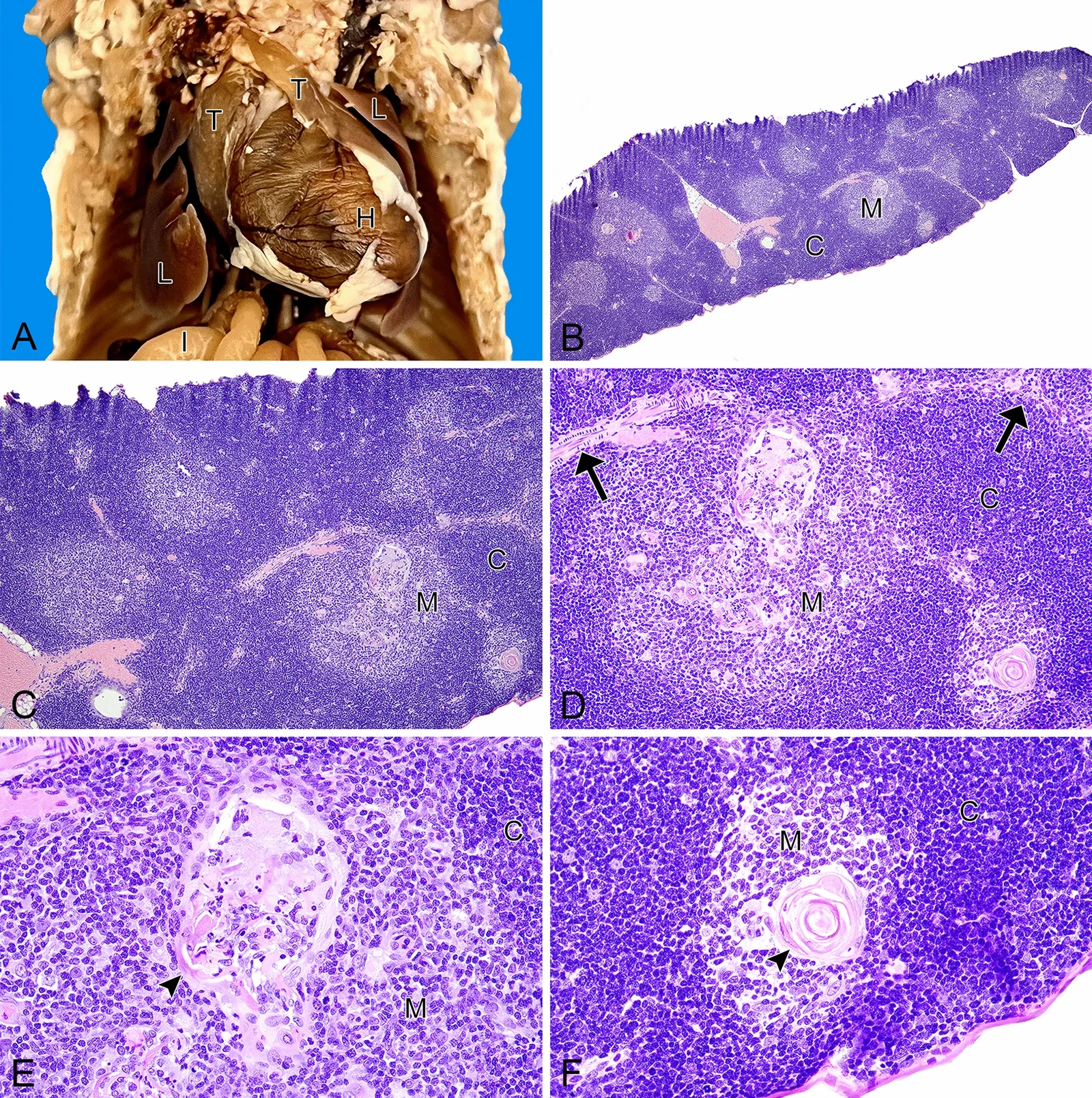
**Gross and histologic features of the thymus in a 6-month-old Egyptian rousette (*Rousettus aegyptiacus*)**. **A** Gross ventrodorsal view of the thoracic cavity showing the bilobed thymus (T) in the pericardial mediastinum. Additionally visible are the heart (H), lungs (L), and intestines (I). Overlying ribs, sternum, and within the abdominal cavity, liver, and diaphragm, have been removed. **B**, **C** Single thymic lobe showing the relative amounts of cortex (C) and medulla (M). The ERB thymus is partially subdivided into indistinct lobules by thin bands of connective tissue that are continuous with the thin connective tissue capsule. Tissue chattering at the top of the organ is an artifact of processing. **B** 4×, HE. **C** 10×, HE. **D** Corticomedullary junction containing arterioles with scant perivascular connective tissue. 20×, HE. **E** A large Hassall’s corpuscle (arrowhead) in the thymic medulla containing numerous epithelial cells with large nuclei, degenerative, eosinophilic, granular cytoplasm, and scant cytoplasmic keratinization admixed with cellular debris. Note the prominent cellularity difference between the epithelial cells within the medulla and the small lymphocytes that predominantly compose the cortex. 40×, HE. **F** Another example of a Hassall’s corpuscle containing whorls of keratin. 40×, HE.

The thymus, functioning as a specialized primary lymphoid organ, serves as the exclusive anatomical site for T-cell development, maturation, and central tolerance induction. This bilobed structure consists of two anatomically distinct lobes connected by a connective tissue isthmus, positioned within the pericardial mediastinum with species-specific variations in cervical extension (Figure 2A) [29, 30]. The thymic architecture comprises structurally distinct cortical and medullary compartments separated by the corticomedullary zone, collectively housing developing T-cell populations, epithelial cells, dendritic cells, macrophages, and variable B cell populations (Figure 2B, C) [29, 30].

Thymic arterial supply enters at the corticomedullary junction, subsequently forming elaborate cortical capillary arcade networks that, in conjunction with epithelial and macrophage cellular components, establish the blood–thymus barrier—a specialized microenvironment that restricts systemic antigen exposure during T-cell development [29, 30]. In contrast, medullary capillaries demonstrate fenestrated morphology with enhanced permeability characteristics. The thymus lacks afferent lymphatic drainage; however, efferent lymphatic vessels provide drainage pathways to regional lymph nodes [29, 30].

The thymic cortex contains densely packed populations of immature T cells interspersed with sparse epithelial cells and specialized macrophages responsible for phagocytosing apoptotic lymphocytes during selection processes [29, 30]. Rapidly proliferating lymphoblasts demonstrate characteristic subcapsular localization patterns. The corticomedullary junction harbors arteriolar structures, perivascular B cell populations, plasma cells, and antigen-presenting dendritic cells [29, 30]. The medulla exhibits reduced cellular density compared with cortical regions and contains mature T cell populations, prominent epithelial cells, macrophages, dendritic cells, and distinctive Hassall's corpuscles—specialized keratinized epithelial aggregates involved in T cell regulatory processes (Figure 2D–F) [29, 30].

Prothymocytes represent immature T cell progenitor populations recruited from bone marrow hematopoietic stem cells to the thymus, entering through the corticomedullary junction. These precursor cells undergo sequential stages of differentiation, clonal expansion, selection, and functional maturation as they migrate from cortical to medullary compartments before entering systemic circulation as mature T cells destined for secondary lymphoid organ colonization [29, 30]. This developmental process involves positive selection mechanisms ensuring T cell recognition of self-major histocompatibility complex (MHC) molecules, while negative selection processes eliminate potentially autoreactive clones through apoptotic mechanisms, thereby establishing central immunological tolerance [29, 30].

Age-related reductions in thymic cellularity are classified as physiological involution, whereas comparable cellular decreases resulting from malnutrition, stress responses, or toxic exposures constitute thymic atrophy [31]. Histologically, both processes demonstrate similar morphological characteristics, including cortical lymphocyte depletion and lobular architectural shrinkage. Stress-related and toxic insults can exacerbate or superimpose upon normal age-associated lymphocyte loss, rendering differentiation between atrophy and involution particularly challenging in geriatric specimens [31]. Thymic lesions documented in laboratory animal models include epithelial hyperplasia, neoplastic processes including thymoma and thymic lymphoma, and decreased cellular density attributable to nutritional deficiencies, stress responses, steroid hormone alterations, or immunotoxic exposures [31].

### Lymph nodes

**Figure 3:**
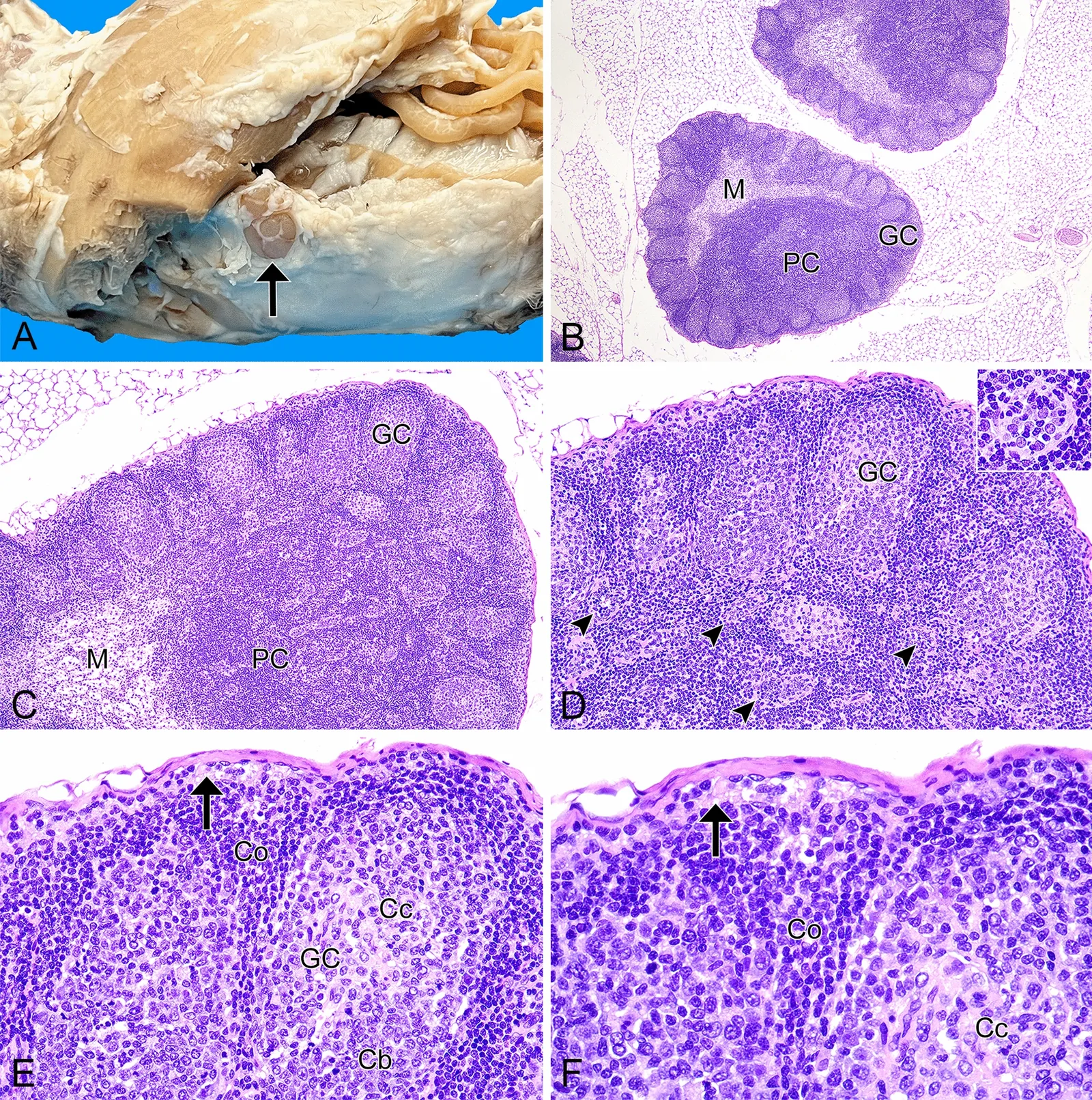
**Gross and histologic features of the lymph node in a 6-month-old Egyptian rousette (*Rousettus aegyptiacus*)**. **A** Right lateral view of an ERB in dorsal recumbency, showing the location of the axillary lymph node (arrow). The right humerus has been removed. **B**, **C** Cross section of an axillary lymph node, showing the cortex containing numerous secondary follicles with germinal centers (GC), robust paracortex (PC), and medulla (M). **B** 4×, HE. **C** 10×, HE. **D** Numerous arterioles lined by high endothelial venules are present within the corticomedullary junction (arrowhead) Inset: higher magnification image of a high endothelial venule. 60×, HE. **E** Proliferating B-cell precursors within germinal centers of secondary follicles displace mature B-cells to the periphery, where they form a basophilic mantle or corona (Co). Mature germinal centers may have a basal population of large, densely packed centroblasts (Cb) and an apical population of smaller, less densely packed centrocytes (Cc). Apical to the secondary follicles is the subcapsular sinus (arrow). 40×, HE. **F** Higher magnification of **E**. 60×, HE.

Lymph nodes represent strategically positioned secondary lymphoid organs distributed along lymphatic vessel networks, serving as critical sites for antigen surveillance and adaptive immune response initiation (Figures 3A, 10A, B) [32, 33]. The functional unit of lymph node architecture is the lymphoid lobule, organized into three anatomically and functionally distinct regions: the B-cell-enriched follicular cortex, the T-cell-predominant paracortex (deep cortical unit), and the medullary compartment (Figure 3B, C) [32]. Lymph node architecture is supported by an intricate, porous reticular meshwork composed of stellate, spindle-shaped, and elongated fibroblastic reticular cells (FRCs) and their associated reticular fiber networks [33]. This structural framework subdivides lymphoid lobules into numerous narrow channels and microenvironmental spaces occupied by lymphocytes, macrophages, and antigen-presenting cells, which typically obscure the underlying architectural meshwork [32, 33].

Lymphocyte entry into lymph nodes occurs via high endothelial venules (HEVs), specialized vascular structures located within interfollicular cortical and paracortical regions that regulate immune cell trafficking through expression of specific adhesion molecules (Figure 3D) [26, 32, 33]. Following extravasation, B cells demonstrate preferential homing to superficial primary cortical follicles where they survey follicular dendritic cells (FDCs) presenting antigen–antibody immune complexes [32, 33]. Upon antigen recognition, B cells undergo clonal expansion and proliferative responses, forming distinctive central germinal centers surrounded by darker mantle zones of displaced resting B cells, thereby transforming primary follicles into secondary follicular structures (Figure 3B–F) [32, 33]. Germinal center development follows predictable temporal patterns, developing within 3–4 days following antigen exposure, achieving peak activity at 7–10 days, and declining in the absence of continued antigenic stimulation [32, 33]. Activated B cell populations differentiate into memory B cells or plasma cell precursors, with the latter undergoing final maturation during migration to medullary cord regions where they function as antibody-secreting effector cells, releasing immunoglobulins into efferent lymphatic circulation [32, 33].

Following HEV-mediated entry, T cells demonstrate preferential homing to paracortical regions where they engage in critical interactions with dendritic cell populations [26]. Dendritic cells function as highly efficient antigen-presenting cells originating from bone marrow hematopoietic stem cells and maintain widespread tissue distribution, with particular concentration at external barrier sites including cutaneous and mucosal surfaces [32, 33]. Upon antigen encounter, dendritic cells migrate to draining lymph nodes via afferent lymphatic vessels and localize within paracortical regions to present processed antigens to T-cell populations, thereby promoting T-cell activation and clonal expansion [32, 33]. Stimulated and proliferating T lymphocytes cause paracortical enlargement but do not generate organized structures analogous to B cell germinal centers [32, 33].

Comprehensive gross anatomical characterization of ERB lymph node distribution has been previously documented, with anatomical locations and numerical ranges as follows: superficial cervical (1–2), facial (2–4), internal jugular (1–3), posterior cervical (1–4), brachial (1–3), axillary (1–4) (Figure 3A), inguinal (2–6), popliteal (2–3), gluteal (2–5), iliac (1–4), renal (1–3), and cranial mesenteric (1–5) [11, 34]. Pregnant ERBs demonstrate increased lymph node numbers and dimensions compared with non-pregnant females or males, with typically larger left-sided lymph nodes [34]. These findings may represent pregnancy-associated immunologic remodeling of preexisting lymphoid tissues, including changes in size and cellular composition of uterine-draining and peripheral lymph nodes, rather than development of additional lymph nodes [35].

Normal histological presentations within lymph nodes include considerable morphological variation reflecting functional adaptation to local antigenic environments. Sinus histiocytosis represents a normal finding in mesenteric lymph nodes, with macrophages potentially containing endogenous pigments (hemosiderin, lipofuscin) or exogenous pigments reflecting antigen uptake from gastrointestinal sources [32, 36]. Mandibular lymph nodes experience continuous antigenic exposure from oropharyngeal regions and characteristically demonstrate well-developed secondary follicular structures and substantial plasma cell populations within medullary cords, whereas lymph nodes with limited antigenic exposure typically maintain primary follicular architecture. Common pathological findings documented in laboratory animal models include lymphoid necrosis, lymphatic sinus ectasia, vascular alterations including sinus congestion and erythrocytosis, nodal and perinodal angiectasia, hemorrhagic changes, vascular proliferation, intracellular pigment accumulation (hemosiderin or ceroid/lipofuscin), amyloidosis, lymphadenitis, lymphocyte hyperplasia, plasma cell hyperplasia, macrophage hyperplasia, lymphomatous processes, and metastatic lesion development [36].

### Spleen

The spleen represents the most substantial secondary lymphoid organ within the mammalian immune system, harboring approximately 25% of the body’s total lymphocyte population and serving as the primary site for blood-borne immune surveillance and adaptive immune responses [37, 38].

This organ orchestrates multiple critical physiological functions, including the systematic filtration of circulating foreign material, the selective removal of senescent erythrocytes, and the coordination of systemic immunological responses to blood-borne antigens. The splenic architecture comprises two distinct compartments: the white pulp, which is the lymphoid component involved in adaptive immunity, and the red pulp, which functions in filtration, iron storage, and hematopoiesis (in rodents and neonates) [37, 38].

The spleen exhibits a distinctive structural organization characterized by encapsulation within a robust fibroelastic capsule reinforced with smooth muscle trabeculae that provide both structural integrity and contractile functionality [37, 38]. Unlike conventional lymphoid organs, the spleen lacks afferent lymphatic drainage and instead maintains direct integration with systemic circulation through specialized vascular arrangements that facilitate continuous blood filtration and immune surveillance.

Blood enters through the splenic artery and subsequently branches into progressively smaller trabecular and central arterioles before ultimately distributing through two distinct circulatory pathways [37, 38]. Approximately 90% of splenic blood flow traverses the venous sinuses in a closed circulatory pattern, while the remaining circulation percolates through the reticular meshwork in an open circulation system before eventual drainage into the splenic vein. Owing to this unique vascular architecture, spleens do not have high endothelial venules, distinguishing splenic lymphocyte trafficking patterns from those observed in conventional lymph nodes [39].

The red pulp constitutes the primary filtration compartment of the spleen, consisting of specialized splenic cords and venous sinuses that form an intricate macrophage-enriched reticular network optimized for the selective removal of aged erythrocytes and circulating particulate matter [37, 38]. This compartment maintains a diverse cellular population, including resident erythrocytes, granulocytes, and circulating mononuclear cells distributed throughout the intercordal spaces, alongside dynamic populations of lymphocytes, hematopoietic precursors, plasma cells, and plasmablasts that migrate from activated follicular and periarteriolar lymphoid sheath regions following antigen-specific differentiation processes [37, 38]. The red pulp serves as a critical component of systemic homeostasis through its specialized filtration capabilities and selective cellular processing functions. The macrophage-rich environment facilitates efficient recognition and phagocytosis of senescent erythrocytes, while simultaneously providing essential iron recycling mechanisms and maintaining optimal circulating blood cell populations.

The white pulp, supported by specialized reticular frameworks that facilitate optimal lymphocyte interactions and antigen presentation processes, consists of the periarteriolar lymphoid sheath (PALS), follicles, and the marginal zone [37, 38]. The periarteriolar lymphoid sheath (PALS) exhibits distinct compartmentalization, featuring an inner T-cell-dominant region populated primarily by CD4 + and CD8 + T lymphocyte subsets interspersed with professional antigen-presenting dendritic cells, and an outer region containing mixed populations of B and T lymphocytes, macrophages, and antibody-secreting plasma cells. [37, 38]. B-cell-enriched follicles are continuous with PALS and demonstrate remarkable plasticity in response to antigenic stimulation, developing germinal center structures that serve as sites of B-cell proliferation, somatic hypermutation, and affinity maturation processes [37, 38]. These dynamic structures represent critical sites for the generation of high-affinity antibody responses and the establishment of immunological memory.

The marginal zone occupies a strategically positioned interface between the red and white pulp compartments, functioning as a specialized antigen-screening environment that facilitates continuous surveillance of systemic circulation [37, 38]. This region maintains distinct cellular populations, including marginal zone B cells with unique antigen recognition capabilities, specialized macrophage subsets, fibroblasts, and dendritic cells that collectively orchestrate rapid responses to blood-borne antigens [37, 38]. The marginal zone boundaries are demarcated by specialized metallophilic macrophages and the marginal sinus, which separate this critical surveillance zone from the PALS and follicular regions [37, 38].

**Figure 4:**
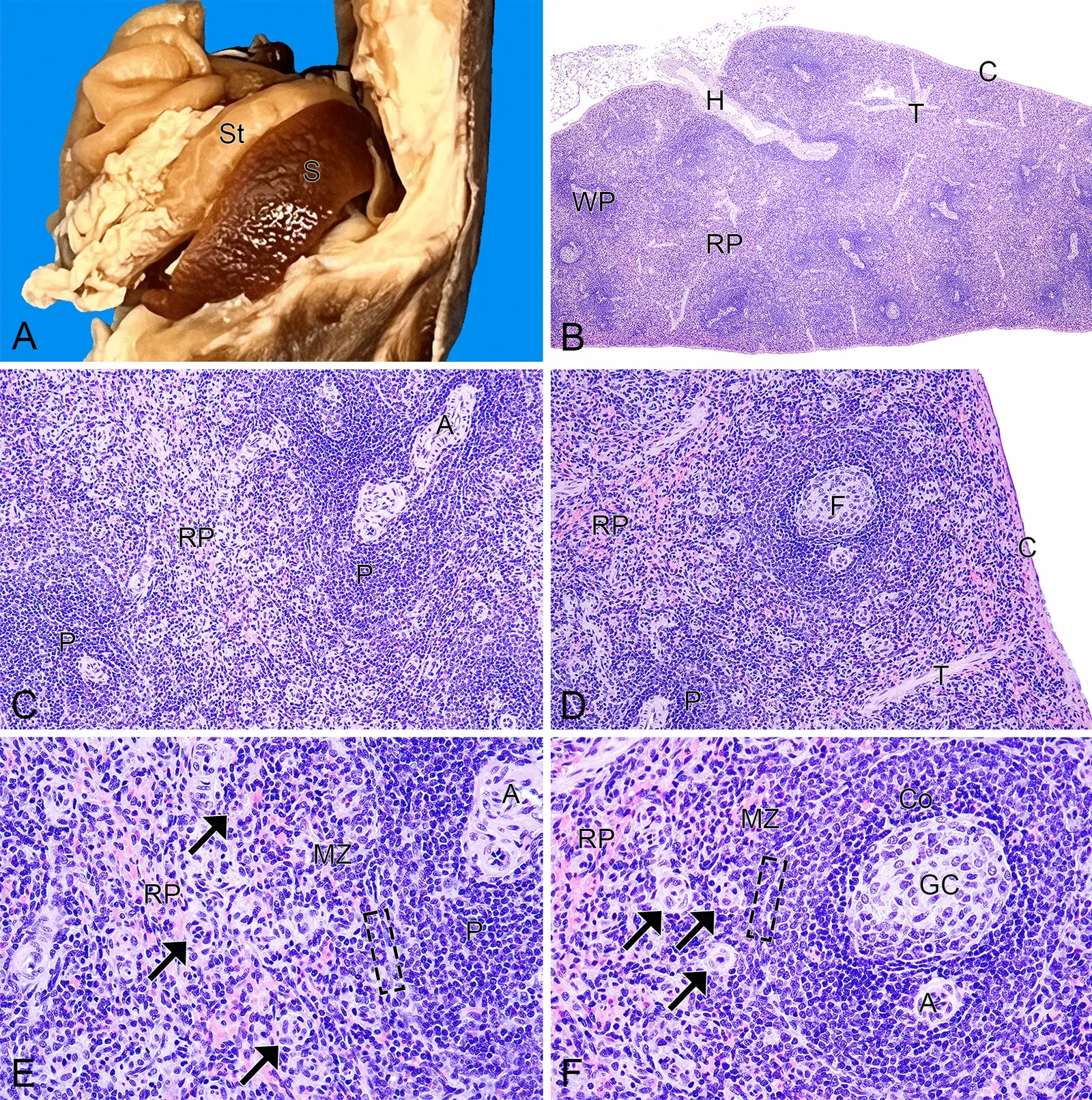
**Gross and histologic features of the spleen in a 6-month-old Egyptian rousette (*Rousettus aegyptiacus*)**. **A** Left lateral view of an ERB, showing the location of the spleen (S) along the greater curvature of the stomach (St). The liver and left lateral flank have been removed. **B**–**F** Cross sections of the spleen showing the splenic hilus (H), white pulp (WP), red pulp (RP), capsule (C), trabeculae (T), central arteries (A), periarteriolar lymphoid sheaths (P), white pulp follicles (F) with germinal centers (GC), surrounding mantle zone/corona (Co), marginal sinus (rectangular box with dashed lines), marginal zone (MZ), and clusters of ellipsoids (arrow). **B** 4×, HE. **C**, **D** 20×, HE. **E**, **F** 40×, HE.

Egyptian rousette bat splenic architecture is conserved with human and rodent organizational patterns, characterized by a thin but functionally robust capsule and variably sized collagenous and smooth muscle trabeculae that provide appropriate structural support (Figure 4A, B) [40]. However, detailed histological analysis of *Rousettus* species reveals distinctive cellular composition patterns, including notably reduced erythroid and myeloid cell populations within the red pulp, absence of megakaryocytes, and exceptionally prominent marginal zone development within the white pulp architecture (Figure 4C–F) [40].

Rousettus species demonstrate unique architectural specializations, including well-developed reticular networks that establish direct connections between marginal zones and periarteriolar lymphoid sheaths, as documented in *Rousettus leschnaultii* bats [41]. These specialized structures, designated as marginal zone bridging channels, are hypothesized to facilitate efficient lymphocyte trafficking from marginal zone surveillance sites to  periarteriolar lymphoid sheaths, potentially enhancing the speed and efficiency of adaptive immune responses to circulating antigens.

A distinctive feature of ERB splenic architecture involves the prominent presence of ellipsoids—specialized structures consisting of arterial capillaries surrounded by dense, circumferential clusters of macrophages, also known as periarterial macrophage sheaths or Schweigger-Seidel sheaths (Figure 4E, F) [37]. These ellipsoid structures appear frequently in clusters throughout both red pulp and marginal zone regions, serving critical roles in filtration processes and the capture of circulating particulate material, while also demonstrating capacity for immune complex trapping. Additionally, periellipsoid lymphoid sheaths have been identified in *R. leschnaultii* bats, representing structures previously characterized primarily in species lacking conventional marginal zones (including poultry and reptiles), where they function as specialized blood–spleen barriers [42, 43]. The functional significance of these structures in Rousettus species requires further elucidation.

ERB spleens demonstrate adaptive capacity in response to physiological stress, with documented reduction in overall organ size during periods of environmental or metabolic stress [44]. This adaptive response likely represents an evolutionary optimization for resource conservation during challenging environmental conditions, while maintaining essential immune surveillance functions.

Comprehensive analysis of splenic pathology in laboratory animal models reveals a spectrum of nonproliferative alterations that may occur within the splenic microenvironment, such as atrophy, pigment, parenchymal and capsular fibrosis, lipidosis/fatty infiltration/lipid metaplasia, vacuolization of splenic histiocytes, amyloidosis, splenic necrosis/infarcts, lymphoid necrosis/apoptosis, extramedullary hematopoiesis, focal red pulp hyperplasia, and focal white pulp hyperplasia. Neoplastic lesions such as lymphocyte hyperplasia and lymphosarcoma, various leukemias, histiocytic sarcomas, mast cell tumors, hemangiomas and hemangiosarcomas, and mesotheliomas have also been reported [45].

### Mucosa-associated lymphoid tissue

Mucosa-associated lymphoid tissue (MALT) represents a highly specialized and strategically distributed component of the immune system, serving as the primary interface for antigen recognition and immune response initiation along mucosal surfaces throughout the body [26]. This immunological network operates with autonomy from systemic immune mechanisms while harboring approximately half of the organism’s total lymphocyte population, underscoring its fundamental importance in maintaining immunological homeostasis at critical environmental interfaces [46]. The strategic positioning of MALT at mucosal barriers enables continuous surveillance of potential antigenic challenges from the external environment, facilitating rapid and targeted immune responses that protect internal tissues while maintaining tolerance to commensal microorganisms and harmless environmental antigens.

MALT is positioned across multiple organ systems to provide comprehensive mucosal immune coverage. The primary components include gut-associated lymphoid tissue (GALT), which serves as the most extensive mucosal immune network; nasopharynx-associated lymphoid tissue (NALT), providing specialized respiratory tract surveillance; and bronchus-associated lymphoid tissue (BALT), facilitating pulmonary immune responses. Additional specialized MALT components include conjunctiva-associated lymphoid tissue (CALT), lacrimal duct-associated lymphoid tissue (LDALT), larynx-associated lymphoid tissue (LALT), and ductal-associated lymphoid tissue (DALT) within salivary glands, among numerous other anatomically specialized sites that collectively provide comprehensive mucosal immune coverage [46, 47].

Functionally, MALT can be categorized as inductive sites or effector sites. Inductive sites function as primary centers for antigen recognition and lymphocyte activation, where antigen-primed B and T cells undergo initial activation processes and clonal expansion. These sites, exemplified by Peyer’s patches within GALT and NALT structures in rodents and nonhuman primates, contain highly organized lymphoid architectures that facilitate optimal antigen presentation, immunoglobulin (Ig)A class switching, and B-cell clonal expansion following T-cell activation. However, this functional distinction maintains considerable flexibility, as certain lymphoid aggregates, including cryptopatches and lymphocyte-filled villi, demonstrate overlapping roles that require further elucidation [46, 47].

Following activation within inductive sites, lymphocytes undergo systematic migration to effector sites distributed throughout mucosal tissues, where they execute specialized immune functions, particularly secretory IgA (sIgA) production [46, 47]. These effector sites maintain characteristic cellular compositions, predominantly featuring CD4 + T helper cells, IgA-secreting plasma cells, dendritic cells, and specialized macrophage populations that collectively orchestrate mucosal immune responses. Intraepithelial lymphocytes, consisting primarily of CD8 + T cells, occupy strategic positions interspersed among epithelial cells and represent critical components of mucosal immunity, likely entering epithelial compartments following activation within conventional lymph nodes and splenic environments [46, 47].

MALT architecture in most regions shares fundamental compartments: follicles, mantles, germinal centers, interfollicular regions, subepithelial domes, and follicle-associated epithelium (FAE) containing microfold (M) cells [47]. M cells are specialized epithelial cells that transport luminal antigens to underlying immune cells, facilitating antigen recognition [46]. Unlike conventional lymphoid organs, MALT lacks afferent lymphatic drainage and medullary regions, instead relying on HEVs as primary entry points for circulating lymphocyte populations [46, 47].

There is substantial species-specific variation in MALT organization and distribution patterns, particularly within GALT components. Rodent species demonstrate uniformly distributed Peyer’s patches throughout the small intestine, while canine GALT exhibits distinctive organizational patterns featuring two morphologically distinct types: small, discrete patches distributed throughout the jejunum and upper ileum, and a single, prominent ileal patch that circumferentially encompasses the distal ileum [46]. Rhesus macaques display prominently developed ileal Peyer’s patches, whereas baboons demonstrate smaller patches characterized by reduced populations of IgA + centroblasts. Rabbits possess unique anatomical specializations including the sacculus rotundus and appendix, both structures exceptionally rich in organized lymphoid follicles [46].

**Figure 5:**
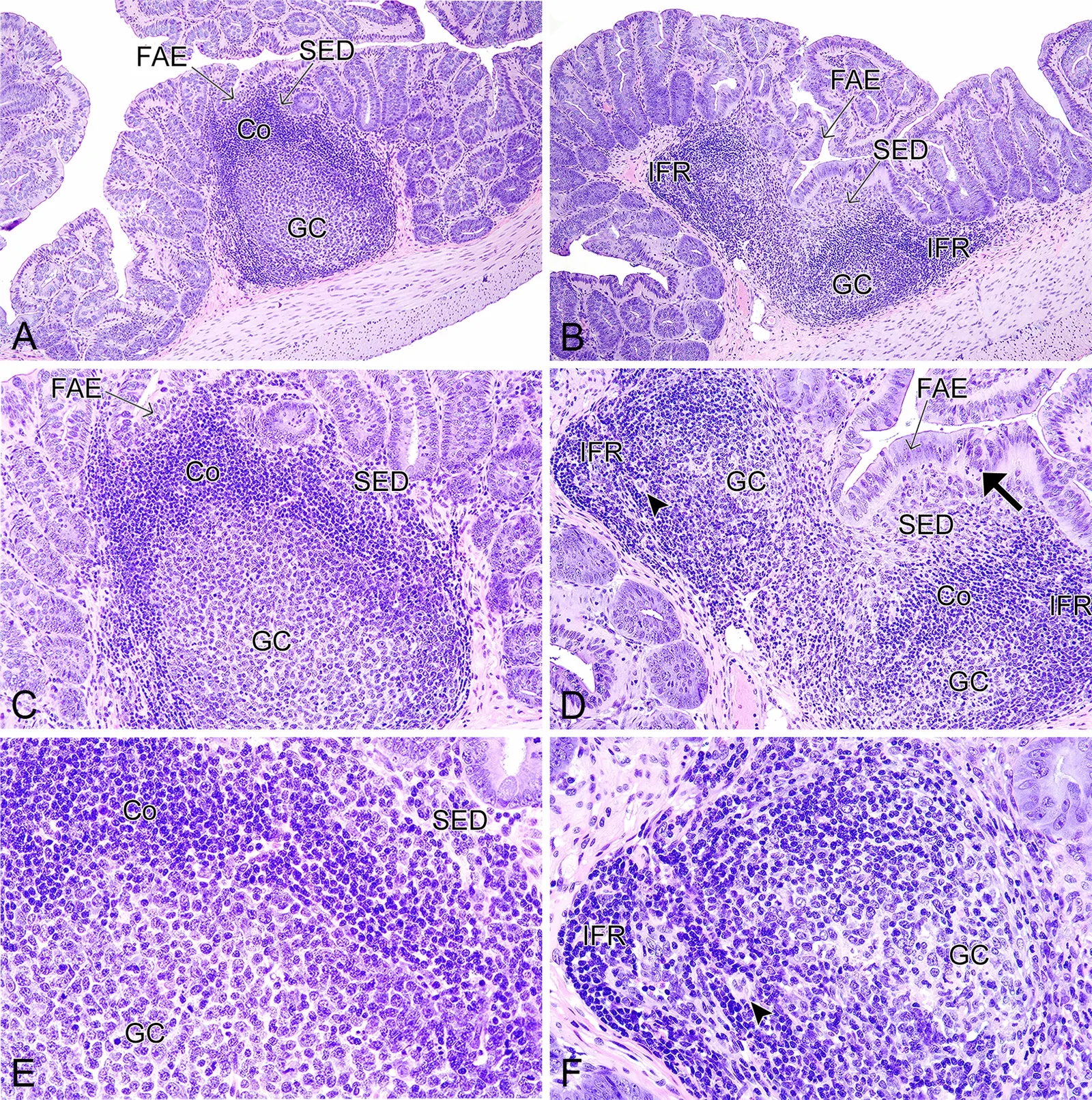
**Histologic features of gastrointestinal-associated lymphoid tissue (GALT) in a 6-month-old Egyptian rousette (*Rousettus aegyptiacus*)**. **A**, **C**, **E** An isolated lymphoid follicle with a germinal center (GC) and overlying mantle zone/corona (Co) expands the lamina propria of the proximal colon. The follicle-associated epithelium (FAE) is separated from the subepithelial dome region (SED). **A** 10×, HE. **C** 20×, HE. **E** 40×, HE. **B**, **D**, **F** A Peyer’s patch expanding the lamina propria of the proximal colon. Low numbers of intraepithelial lymphocytes (arrow) are present. Interfollicular regions (IFR) contain numerous high endothelial venules (arrowhead). **B** 10×, HE. **D** 20×, HE. **F** 40×, HE.

In ERBs, GALT demonstrates characteristic mammalian organizational patterns and was identified as both isolated lymphoid follicles and more commonly as organized Peyer’s patches distributed throughout the jejunum, ileum, and both proximal and distal colon (Figure 5). These structures, while not grossly appreciable during necropsy examination, demonstrate well-developed microscopic architecture consistent with comprehensive and functional immune surveillance capabilities.

**Figure 6:**
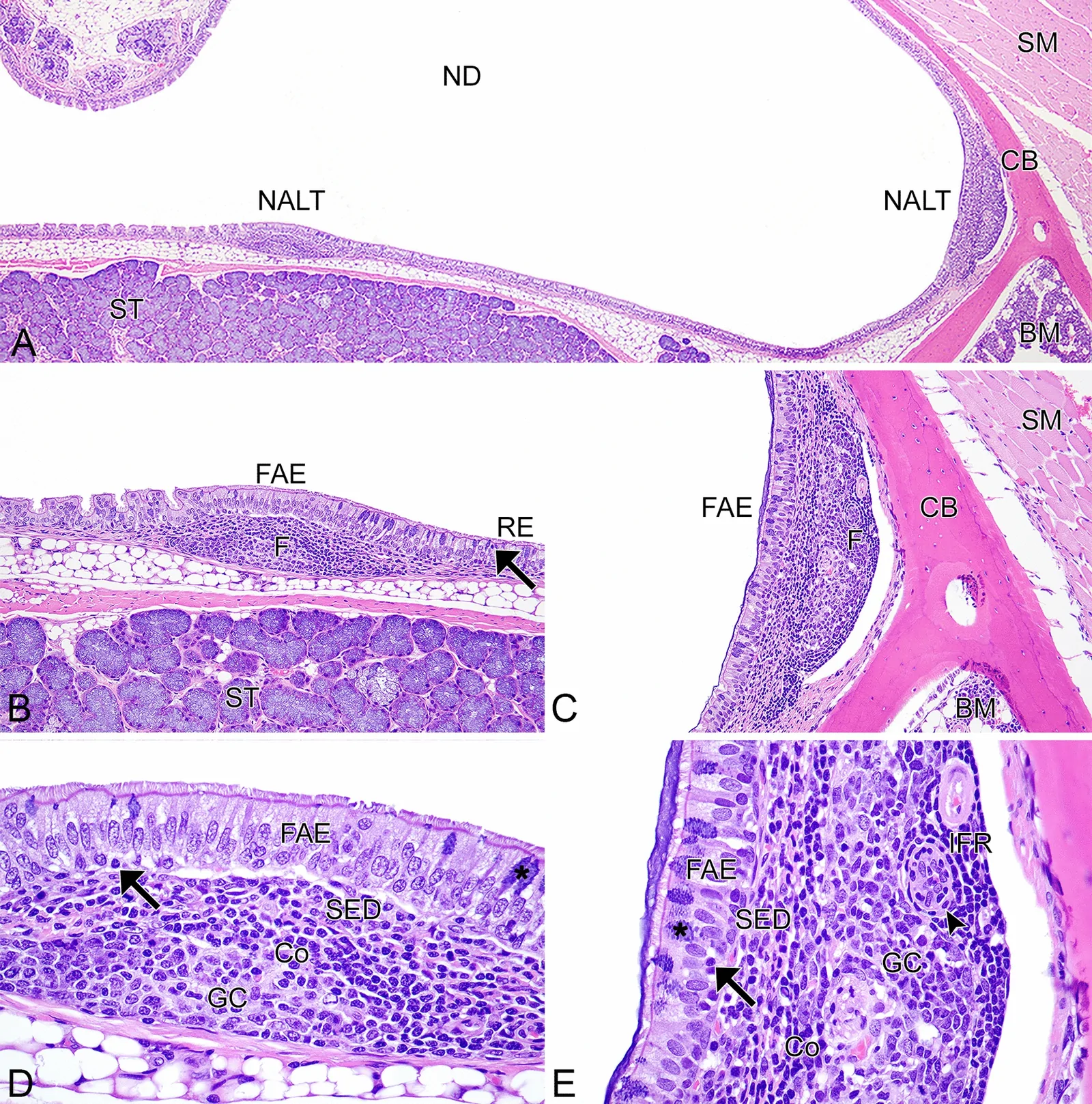
**Histologic features of nasal-associated lymphoid tissue (NALT) in a 6-month-old Egyptian rousette (*Rousettus aegyptiacus*)**. **A** Nasal-associated lymphoid tissue (NALT) on the lateroventral and ventral floor of the proximal nasopharyngeal duct (ND). Salivary tissue (ST) is present between the ND and hard palate (out of frame). BM, bone marrow; SM, skeletal muscle; CB, cortical bone. 4×, HE. **B**, **D** A well-defined NALT follicle (F). Goblet cells (asterisk) and intraepithelial lymphocytes (arrow) are typically absent in the follicle-associated epithelium (FAE) immediately overlying the follicle, however, can be found on the peripheral FAE and throughout normal respiratory epithelium (RE). FAE remains densely ciliated. GC, germinal center; Co, corona/mantle zone; SED, subepithelial dome. **B** 20×, HE. **D** 60×, HE. **C**, **E** A less well-defined NALT follicle. The germinal center (GC), corona/mantle zone (Co), and subepithelial dome (SED) are more difficult to distinguish. Prominent high endothelial venules (arrowhead) are present within the interfollicular region (IFR). There are occasional intraepithelial lymphocytes (arrow) and goblet cells (asterisk) within the follicle-associated epithelium (FAE). **C** 20×, HE. **E** 60×, HE.

NALT, documented across diverse mammalian species including rodents, rabbits, chickens, and nonhuman primates, consists of paired lymphoid aggregates within nasal passages for respiratory immune surveillance (Figure 6) [46, 47]. NALT demonstrates distinctive cellular compositions compared with Peyer’s patches, featuring reduced intraepithelial lymphocyte populations, more balanced B- and T-cell distributions, and decreased dendritic cell concentrations within subepithelial dome regions [46, 47].

**Figure 7:**
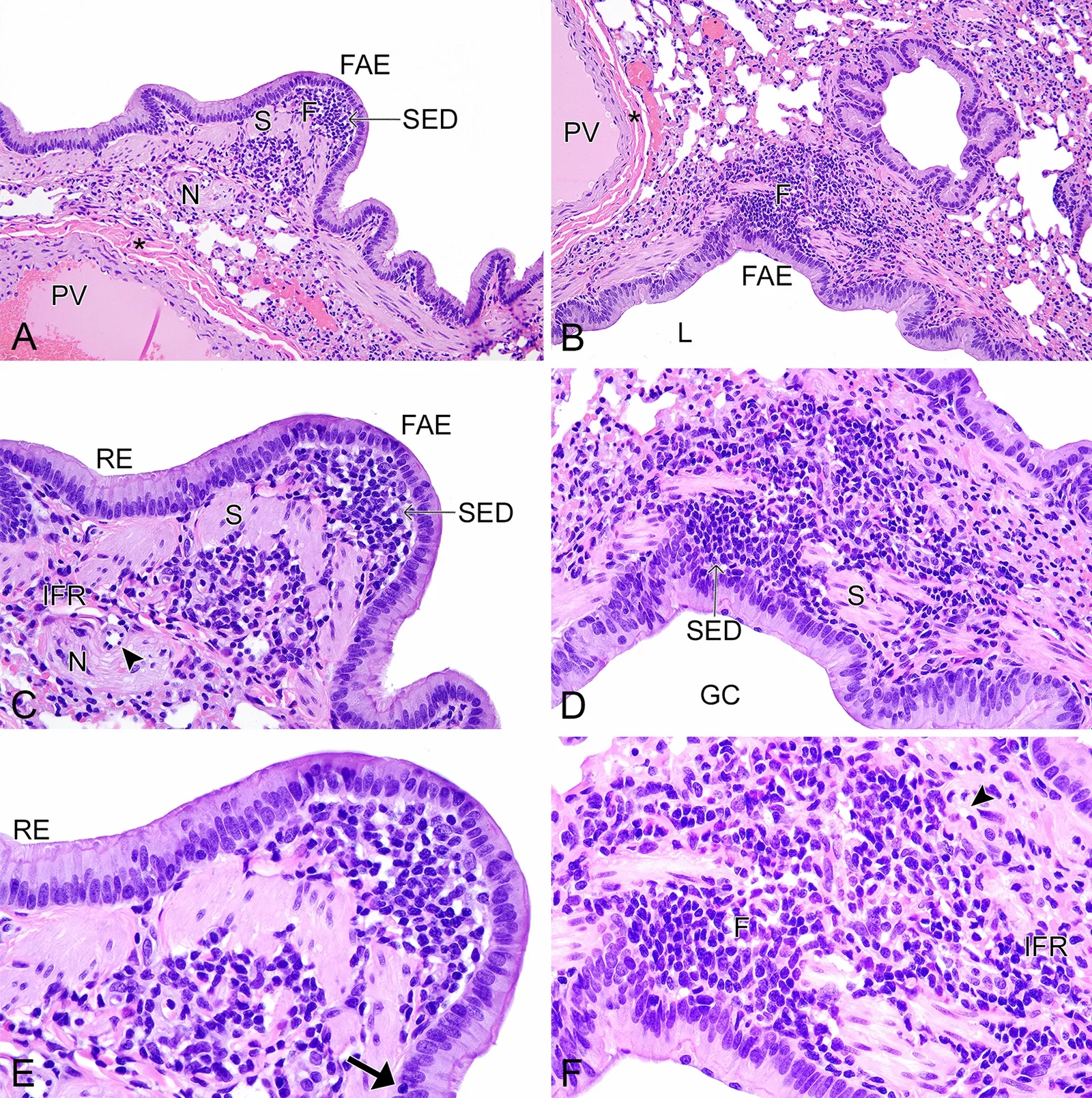
**Histologic features of bronchus-associated lymphoid tissue (BALT) in a 6-month-old Egyptian rousette (*Rousettus aegyptiacus*)**. **A**–**F** Two examples of BALT along a primary bronchiole. Germinal centers within follicles (F) are uncommon, and thin subepithelial domes (SED) and interfollicular regions (IFR) are difficult to distinguish. Overlying follicle-associated epithelium (FAE) generally lacks cilia and goblet cells. The presence of high endothelial venules (arrowhead) may be the only way to distinguish the IFR. N, nerve; PV, pulmonary vein; asterisk, cardiomyocytes surrounding pulmonary vein; S, smooth muscle; arrow, intraepithelial lymphocyte. **A**, **B** 20×, HE. **C**, **D** 40×, HE. **E**, **F** 60×, HE.

BALT is described in a variety of vertebrate species, including mammals, birds, and reptiles. Rabbits and rats typically exhibit the most extensive BALT development, followed by mice and guinea pigs. [47]. Notably, BALT development demonstrates significant dependence on antigenic stimulation, as evidenced by its absence in germ-free pigs, reduction in germ-free rats, and requirement for postnatal antigenic exposure in mice and humans [46, 47]. Structurally, BALT resembles NALT architecture but lacks follicular dendritic cells (FDCs) and has reduced populations of intraepithelial lymphocytes and germinal centers (Figure 7) [46].

**Figure 8:**
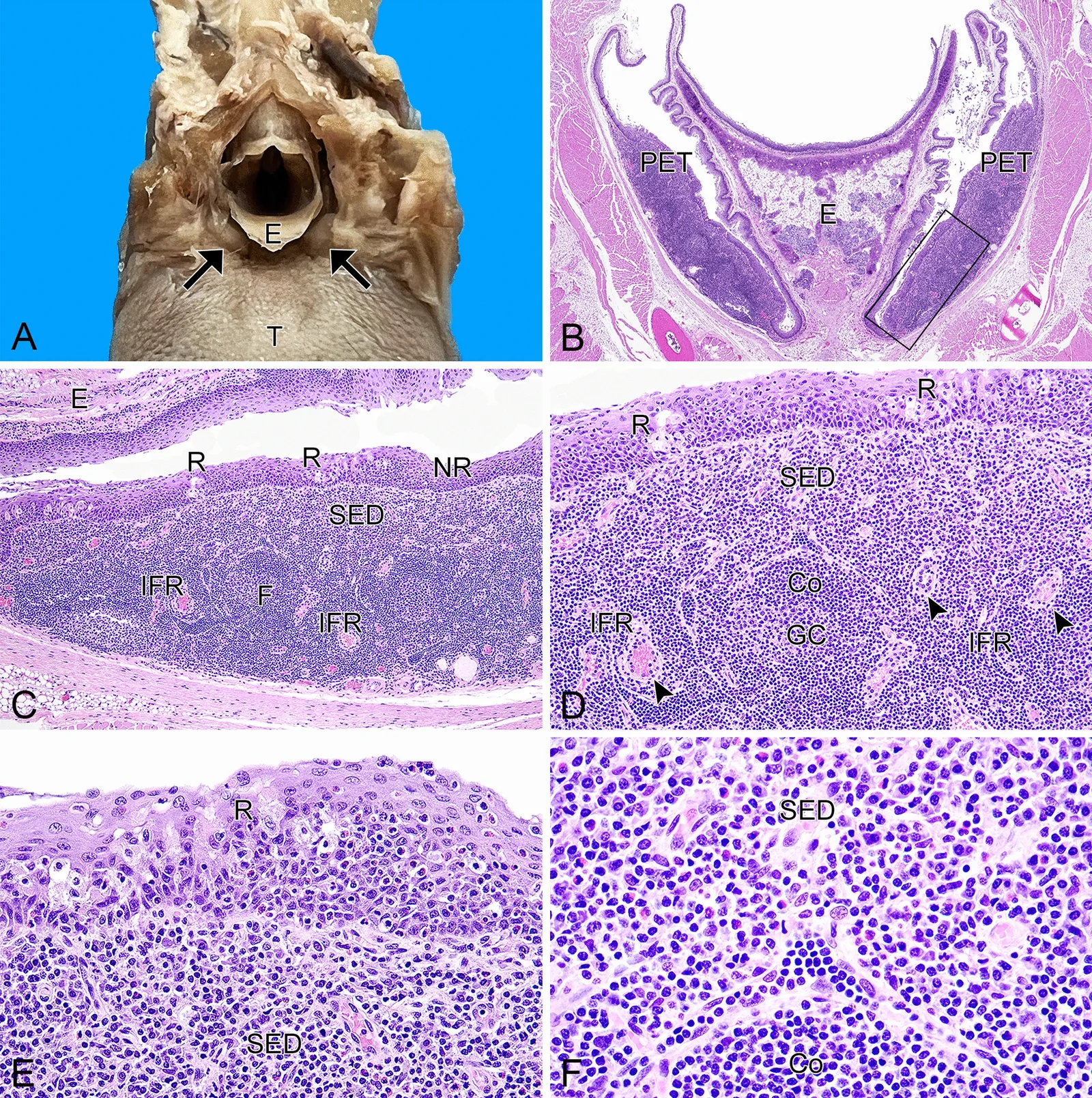
**Gross and histologic features of the paraepiglottic tonsils in a 6-month-old Egyptian rousette (*Rousettus aegyptiacus*)**. **A** Rostral view of the larynx, showing the epiglottis (E), paraepiglottic tonsils (arrows), and tongue (T). The pharyngeal roof, soft palette, and adjacent soft tissue and skeletal muscle have been removed. **B** Transverse section of epiglottis (E) and paraepiglottic tonsils (PET). The area in **C**–**F** is delineated with a black rectangle. 2×, HE. **C**–**F** Reticular (R) and nonreticular (NR) epithelium overlies the paraepiglottic tonsil, which is otherwise structurally similar to a Peyer’s patch. E, epiglottis; SED, subepithelial dome; F, follicle; IFR, interfollicular region; GC, germinal center; Co, corona/mantle zone; R, reticular epithelium; NR, nonreticular epithelium; arrowhead, high endothelial venule. **C** 10×, HE. **D** 20×, HE. **E** 40×, HE. **F** 60×, HE.

**Figure 9:**
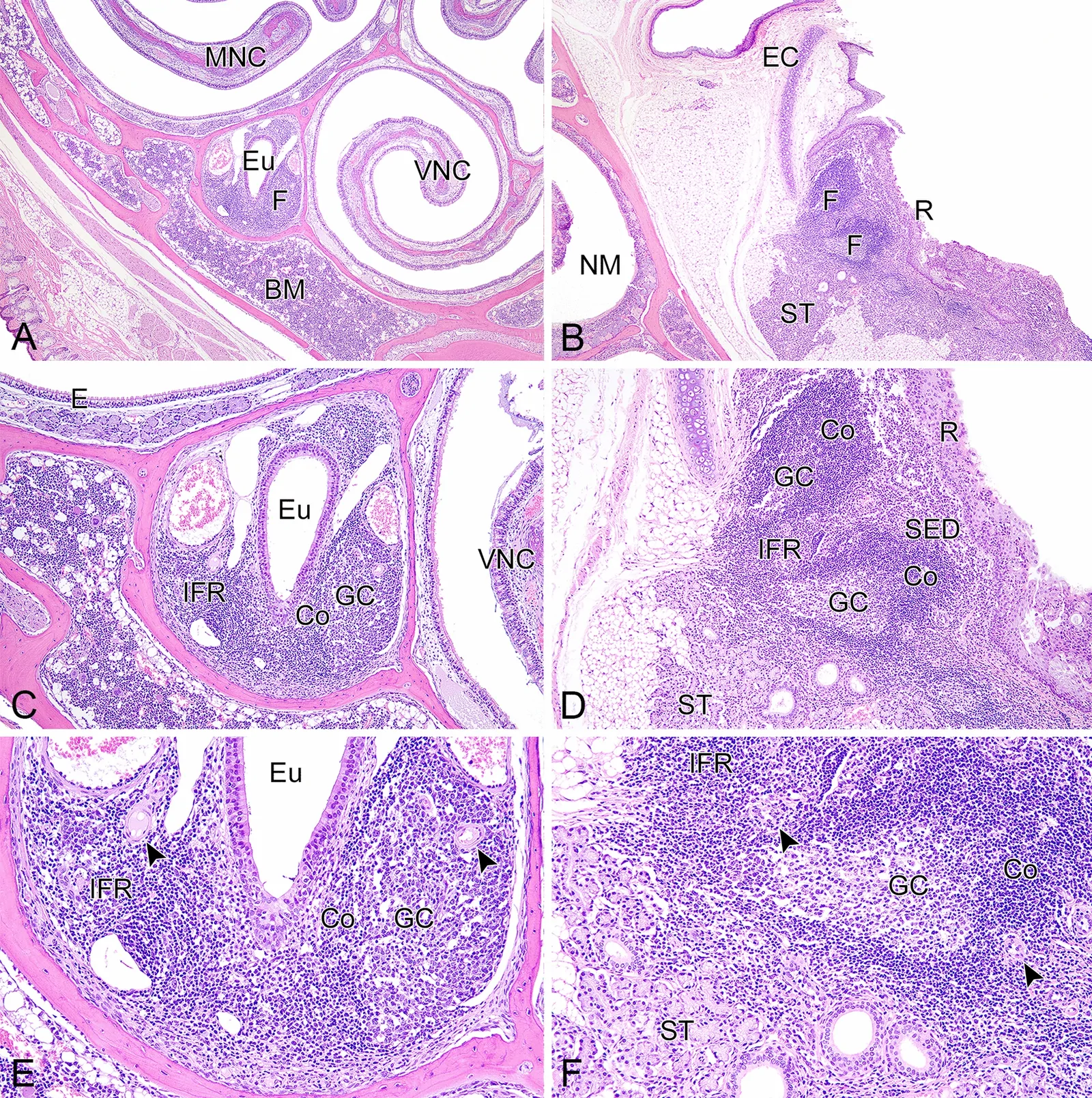
**Histologic features of the tubal tonsil in a 6-month-old Egyptian rousette (*Rousettus aegyptiacus*)**. **A**, **C**, **E** Tubal tonsil surrounding the auditory or Eustachian tube (Eu), with typically ventrolateral follicles (F). MNC, middle nasal concha; VNS, ventral nasal concha; BM, bone marrow; GC, germinal center; Co, corona/mantle zone; IFR, interfollicular region; arrowhead, high endothelial venule. **A** 4×, HE. **C** 10×, HE. **E** 20×, HE. **B**, **D**, **F** Tubal tonsil located at the opening of the auditory tube. EC, elastic cartilage; F, follicle, NM, nasal meatus; ST, salivary tissue; R, reticular epithelium. **B** 4×, HE. **D** 10×, HE. **F** 20×, HE.

Tonsils are absent in rodents but present in most species [46]. Canine species possess four distinct tonsillar types: lingual, palatine, pharyngeal, and tubal tonsils, with morphological variations including crypted (follicular) and non-crypted configurations [46, 47]. Tonsillar crypts function as specialized blind invaginations of surface epithelium extending into submucosal lymphoid tissue, facilitating enhanced antigen capture and processing capabilities [46]. In the current study, palatine tonsils were not definitively identified in histological sections; however, their presence is presumed on the basis of comparative mammalian anatomy and specific documentation in related chiropteran species, including Jamaican fruit bats [48]. While considerable species diversity exists among chiropteran taxa, palatine tonsils likely represent conserved anatomical features that were not captured in the available tissue sections. Similarly, lingual and pharyngeal tonsils were not definitively identified, indicating the need for future comprehensive investigations to fully elucidate MALT components in ERB populations. However, paraepiglottic tonsils were readily identified at the lateral aspects of the epiglottal base (Figure 8), and tubal tonsils were successfully documented adjacent to Eustachian tube openings (Figure 9). These tonsillar structures demonstrate characteristic architecture featuring reticular epithelium containing M cells, lymphocytes, macrophages, and dendritic cells overlying the apices of lymphoid follicles, while nonreticular epithelium covers remaining tonsillar tissue surfaces (Figure 8E) [46].

Comprehensive analysis of MALT pathology in laboratory animal models reveals diverse pathological alterations including inflammatory, degenerative, and neoplastic processes [49]. Inflammatory changes include acute and chronic inflammation, macrophage aggregates, and granulomatous inflammatory responses. Degenerative alterations include tissue degeneration, necrosis, and pathological mineralization. Neoplastic transformations most commonly involve lymphomatous processes, with additional manifestations including neoplastic metastasis and tumor emboli formation within MALT structures. [49].

**Figure 10:**
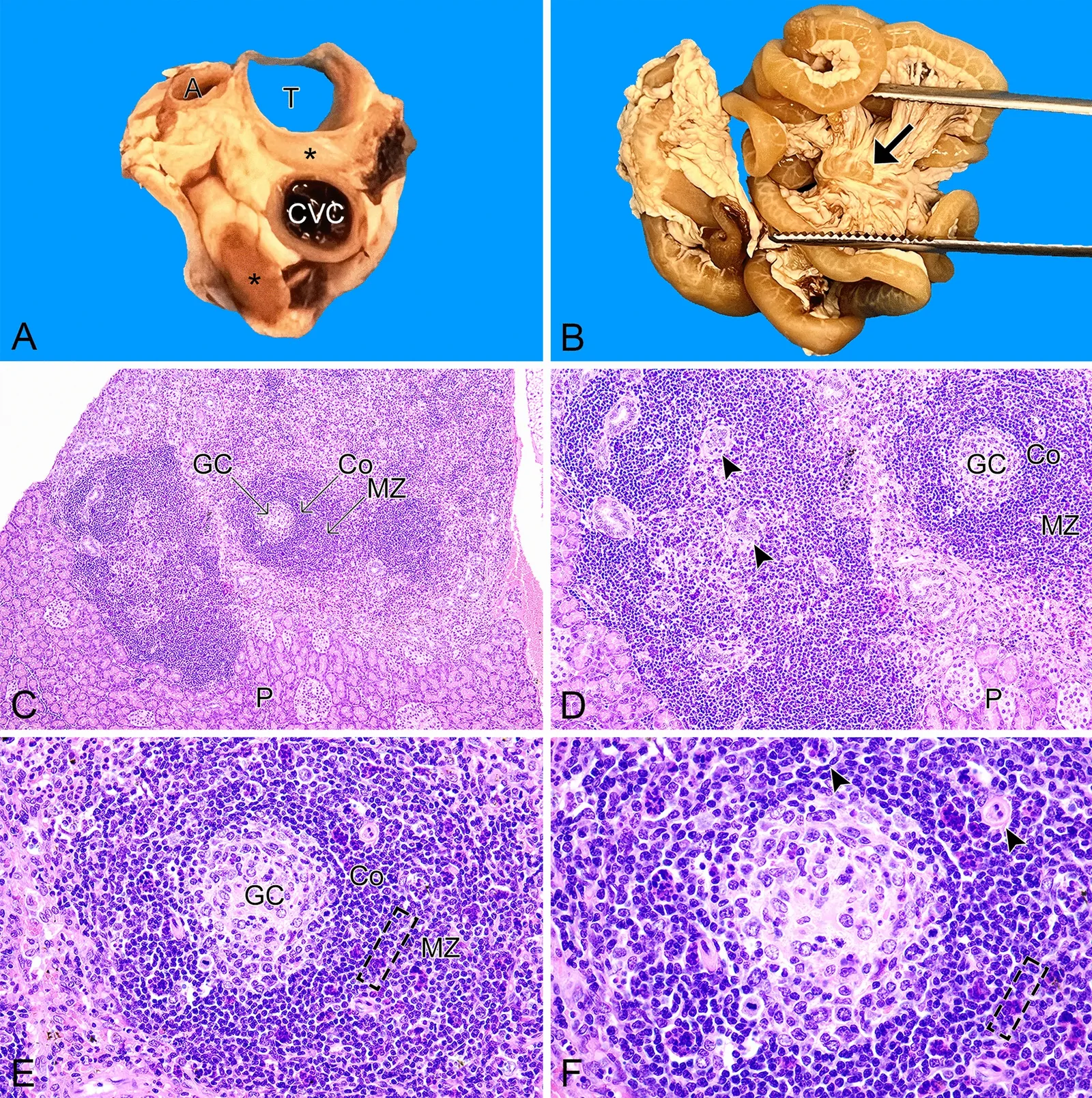
**Additional gross and histologic features of lymphoid tissue in Egyptian rousette (*Rousettus aegyptiacus*)**. **A** Transverse section through thoracic tissue immediately caudal to the aortic arch shows the location of tracheobronchial lymph nodes (asterisk). T, trachea; A, aorta; CVC, cranial vena cava. **B** Cranial mesenteric lymph node (arrow) within the root of the mesentery. **C**–**F** Tertiary lymphoid structures within a locally extensive focus of chronic inflammation in the pancreas of an adult male free-ranging ERB. GC, germinal center; Co, corona/mantle zone; MZ, marginal zone; P, pancreas; black dashed rectangle, marginal sinus; arrowhead, high endothelial venule. **C** 10×, HE. **D** 20×, HE. **E** 40×, HE. **F** 60×, HE.

Of interest in the current study was the identification of tertiary lymphoid structures within pancreatic tissue of an adult male free-ranging specimen, documented here for comprehensive anatomical characterization (Figure 10C–F). Tertiary lymphoid structures, alternatively designated as ectopic lymphoid tissue, represent organized lymphocyte-specific microdomains that develop within non-lymphoid tissues under conditions of chronic inflammatory stimulation [50, 51]. These structures have been documented across diverse mammalian species, including mice, rats, pigs, buffalo, and dogs, typically arising in response to persistent antigenic challenges or chronic inflammatory conditions [52–55].

### Immunohistochemistry

Immunohistochemistry (IHC) represents a sophisticated laboratory technique that exploits specific antigen–antibody interactions to detect and visualize targeted proteins or cellular markers within preserved histological tissue sections. Through the application of antibodies conjugated to enzymes or fluorophores, IHC enables precise spatial localization of target molecules while preserving tissue architecture [56]. This technique is widely used for diagnostic pathology, tumor classification, and infectious disease identification, as well as investigating the distribution patterns of immune cell populations, cytokines, and structural proteins within complex tissue microenvironments.

There are few studies to date that have utilized IHC to investigate ERB diseases. The majority of existing research has concentrated on tumor classification, infectious disease characterization, and immunomodulatory responses within the context of viral infection [7, 57–65]. Despite most commercially available IHCs being produced for use in humans, laboratory animals, or companion animals, investigators have achieved success in demonstrating cross-reactivity with ERB tissues. Table 1 provides comprehensive information regarding primary antibodies that have been employed in immunohistochemical evaluation of ERB tissues, representing the current state of validated reagents for this species.

In the present investigation, several B-cell markers, including CD20, CD21, and Pax-5, proved unsuccessful in achieving specific labeling of their respective target cell populations within ERB tissues. This lack of cross-reactivity likely reflects species-specific differences in epitope structure or post-translational modifications that prevent optimal antibody binding. Additionally, factor VIII-related antigen and smooth muscle actin immunostaining were previously completed as components of unpublished research efforts and are included in Table 1 for comprehensive documentation of validated markers.

Fetal tissues consistently demonstrated absent-to-minimal immunolabeling across all examined lymphoid organs. Fetal cells are often less differentiated and therefore may not yet express target proteins at detectable levels, or may express them as different isoforms or precursor forms that lack recognition by the applied antibodies [56]. Furthermore, epitopes within fetal tissues may not possess the appropriate conformational structure, glycosylation patterns, or post-translational modifications necessary to facilitate optimal antibody binding, even when the target protein is present within the tissue [56]. Additionally, overall tissue quality may have degraded due to extended (~15 year) fixative exposure, which may have rendered tissues less amenable to successful immunohistochemical labeling. Prolonged formalin fixation can result in cross-linking of proteins, masking of antigenic epitopes, and degradation of tissue architecture, all of which can significantly compromise immunohistochemical performance and result in reduced or absent staining intensity.

**Figure 11:**
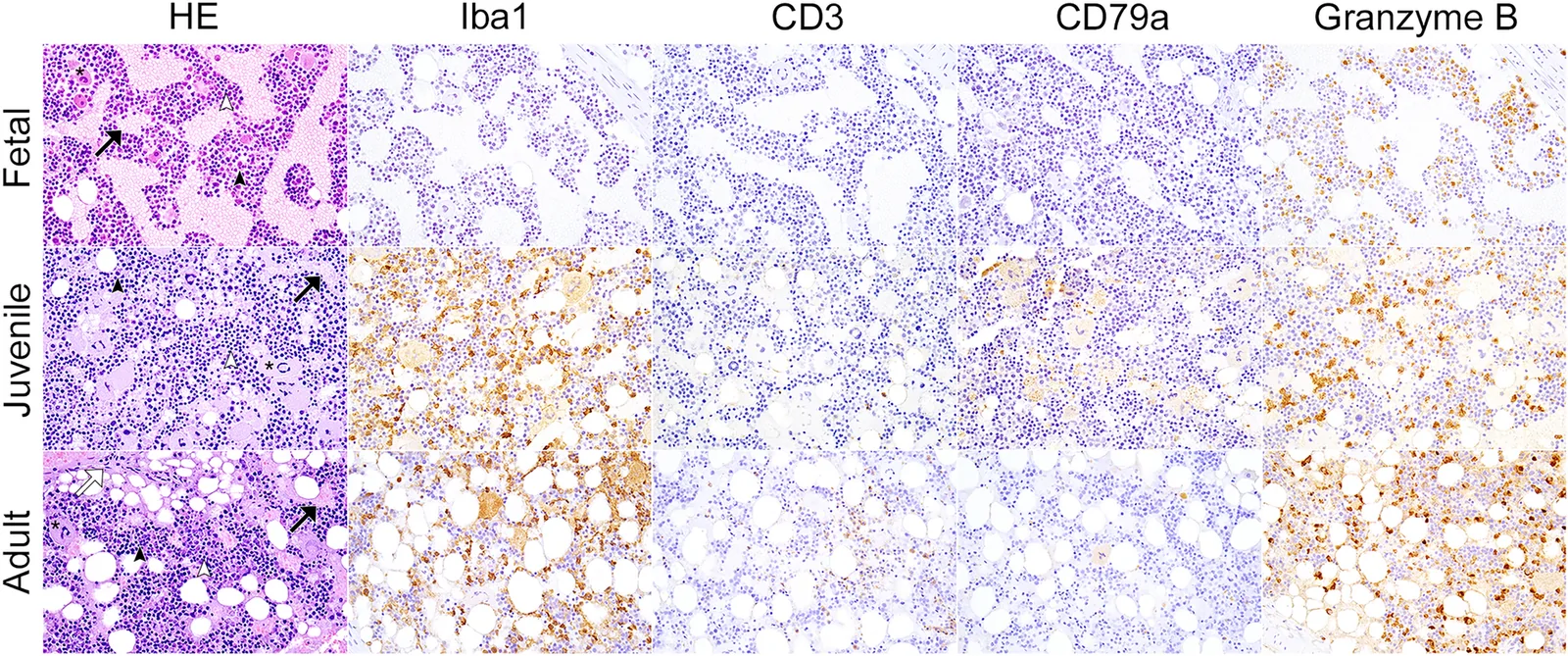
**Comparison of bone marrow immunolabeling across fetal, juvenile, and adult Egyptian rousette (*Rousettus aegyptiacus*) tissues**. Sections are reflective of bone marrow sections within or immediately adjacent to the femoral head; 40× magnification. HE, hematoxylin and eosin; Iba1, ionized calcium-binding adapter molecule 1. Monocyte/macrophage lineage; cytoplasmic reactivity. CD3, cluster of differentiation 3. T-cell receptor complex; membranous or cytoplasmic reactivity. CD79a, cluster of differentiation 79a. B-cell receptor complex; primarily cytoplasmic reactivity, membranous reactivity additionally occurs. Granzyme B: neutral serine proteases in specialized lytic granules in CTLs and NK cells; cytoplasmic reactivity. Fetal bone marrow has positive immunoreactivity with Granzyme B. Juvenile and adult bone marrow has low positive immunoreactivity with CD3 and CD79a, with additional cytoplasmic labeling of megakaryocytes, and robust positive immunoreactivity with Iba1 and Granzyme B. Black arrowhead, myeloid hematopoiesis; white arrowhead, erythroid hematopoiesis; asterisk, megakaryocyte; white arrow, nutrient artery; black arrow, venous sinus.

**Figure 12:**
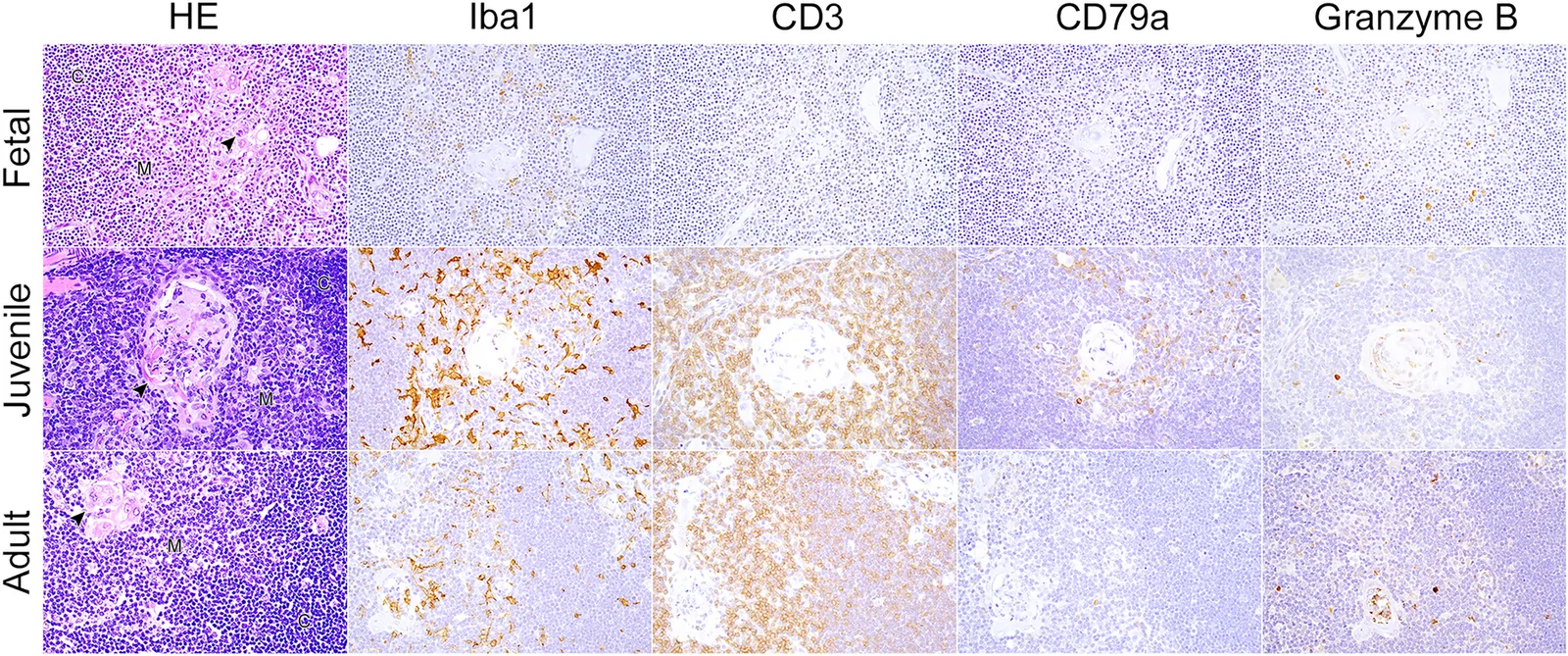
**Comparison of thymic immunolabeling across fetal, juvenile, and adult Egyptian rousette (*Rousettus aegyptiacus*) tissues**. Sections are reflective of the corticomedullary junction, with Hassall’s corpuscles and blood vessels included where possible; 40× magnification. Fetal thymus has scant positive immunoreactivity with CD3 and low positive immunoreactivity with Iba1 and Granzyme B. Juvenile and adult  thymus has low positive immunoreactivity with CD79a and Granzyme B, with additional cytoplasmic labeling of megakaryocytes, and moderate-to-robust positive immunoreactivity with Iba1 and CD3. CD3 immunolabeling highlights the concentration of CD3 + T cells within the thymic medulla. Arrowhead, Hassall’s corpuscle; C, cortex; M, medulla.

**Figure 13:**
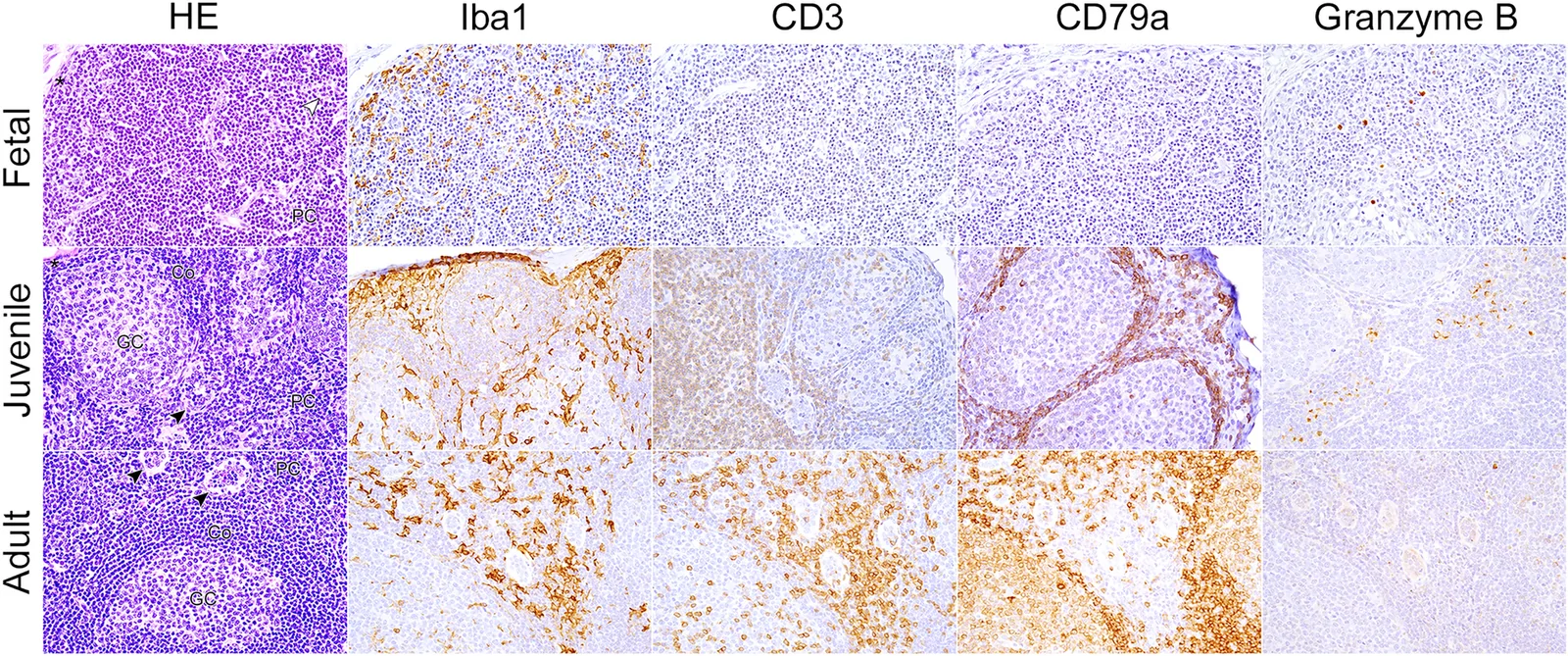
**Comparison of lymph node immunolabeling across fetal, juvenile, and adult Egyptian rousette (*Rousettus aegyptiacus*) tissues**. Sections are reflective of the subcapsular sinus, cortex containing secondary follicles (juvenile and adult), and subjacent paracortex; 40× magnification. Fetal lymph node has faint positive immunoreactivity with CD3 and moderate positive immunoreactivity with Iba1 and Granzyme B. Juvenile and adult lymph node has strong positive immunoreactivity with all stains, highlighting the concentrations of Iba1 + and CD3 + cells within the paracortex, CD79a + cells within the germinal center and corona/mantle zone, and rare Granzyme B + cells within the paracortex. Asterisk, subcapsular sinus; GC, germinal center; Co, corona/mantle zone; PC, paracortex; arrowhead, high endothelial venule.

**Figure 14:**
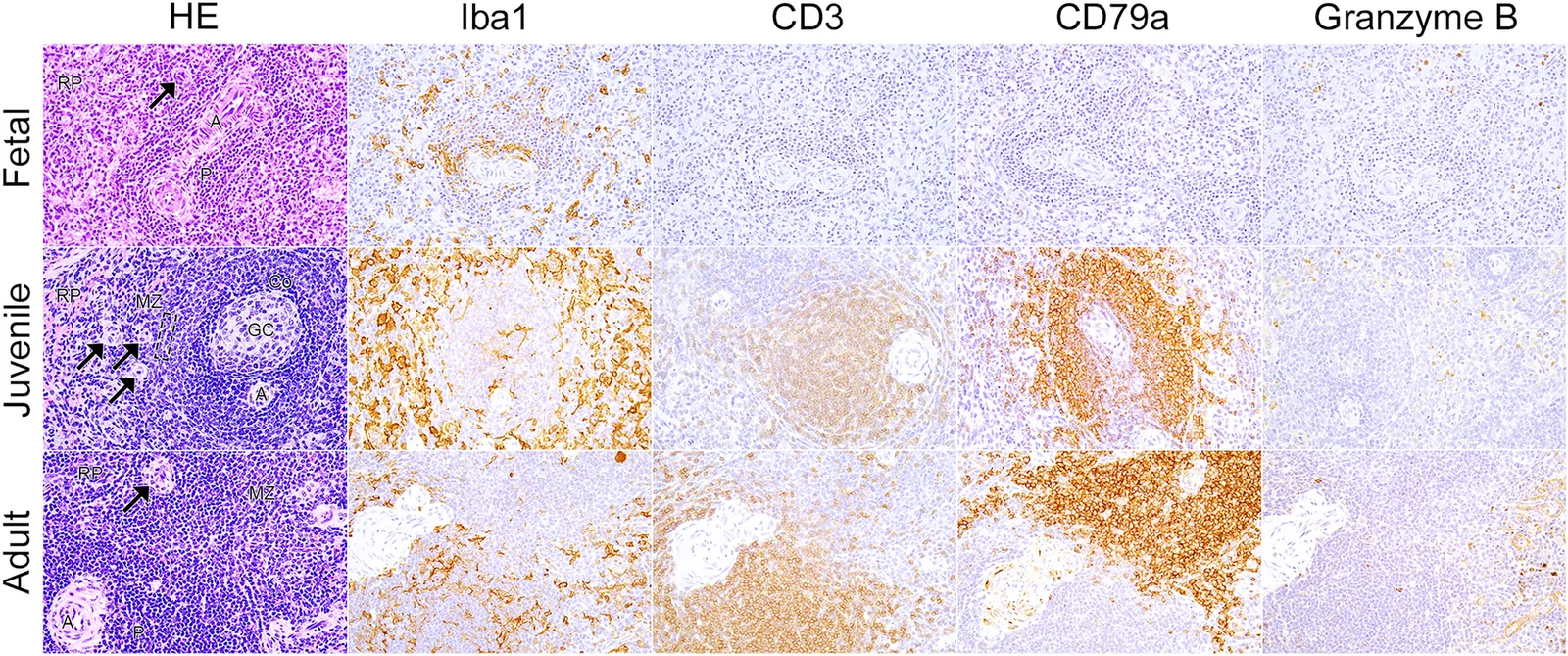
**Comparison of splenic immunolabeling across fetal, juvenile, and adult Egyptian rousette (*Rousettus aegyptiacus*) tissues**. Sections are reflective of white pulp, red pulp, and associated structures; 40× magnification. Fetal spleen has faint positive immunoreactivity with CD3 and CD79a and strong positive immunoreactivity with Iba1 and Granzyme B. Juvenile and adult spleen has strong positive immunoreactivity across all stains, highlighting the concentration of Iba1 + , CD79a + , and Granzyme B + cells within the marginal zone and CD3 + cells within periarteriolar lymphoid sheaths. RP, red pulp; A, central artery; P, periarteriolar lymphoid sheath; GC, germinal center; Co, corona/mantle zone; rectangular box with dashed lines, marginal sinus; MZ, marginal zone; arrow, ellipsoid.

**Figure 15:**
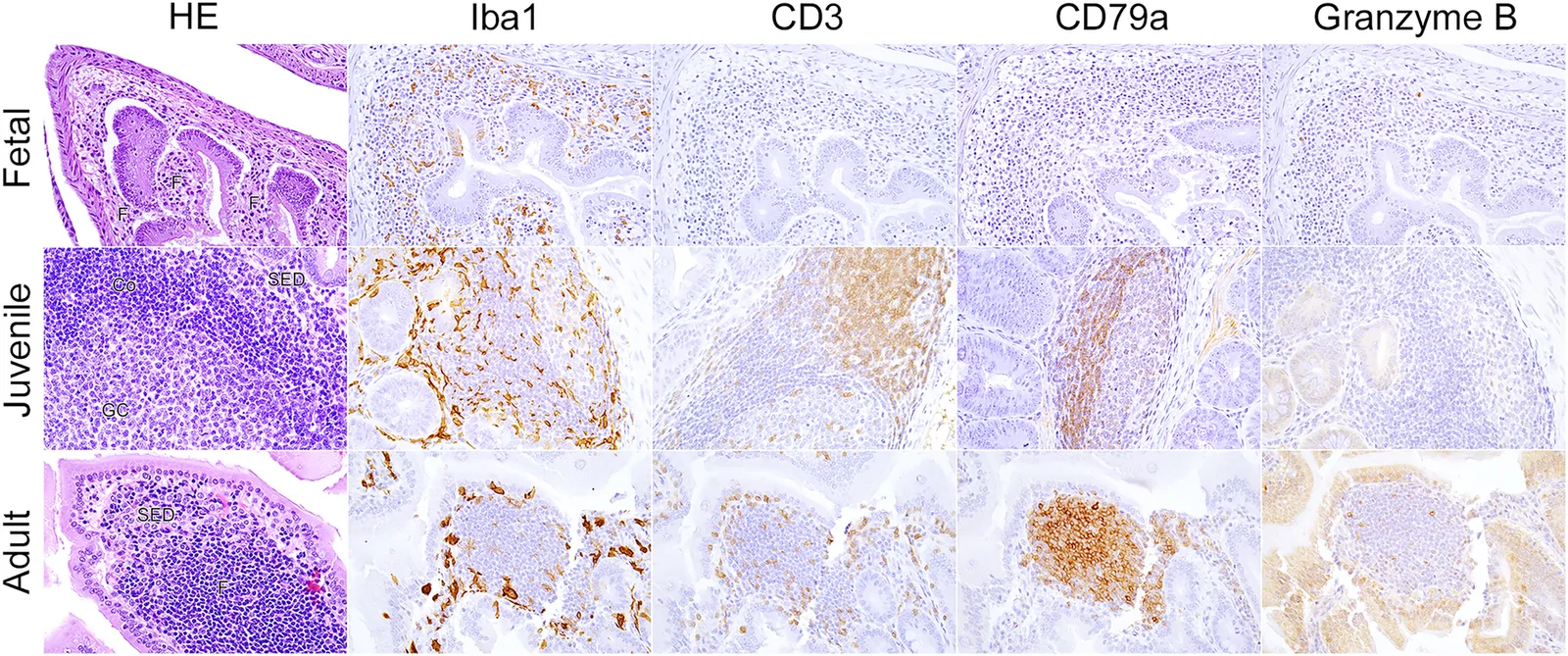
**Comparison of gastrointestinal-associated lymphoid tissue (GALT) immunolabeling across fetal, juvenile, and adult Egyptian rousette (*Rousettus aegyptiacus*) tissues**. Sections are reflective of Peyer’s patches within the proximal colon; 40× magnification. Consistent with other lymphoid organs, fetal GALT has faint positive immunoreactivity with CD3 and CD79a and strong positive immunoreactivity with Iba1 and Granzyme B. Juvenile and adult GALT have strong positive immunoreactivity with all stains except for faint positive immunoreactivity in juvenile tissue with Granzyme B. Adult GALT did not have well-defined germinal centers, likely from its relatively antigen-free managed care environment. F, follicle; GC, germinal center; Co, corona/mantle zone; SED, subepithelial dome.

**Figure 16:**
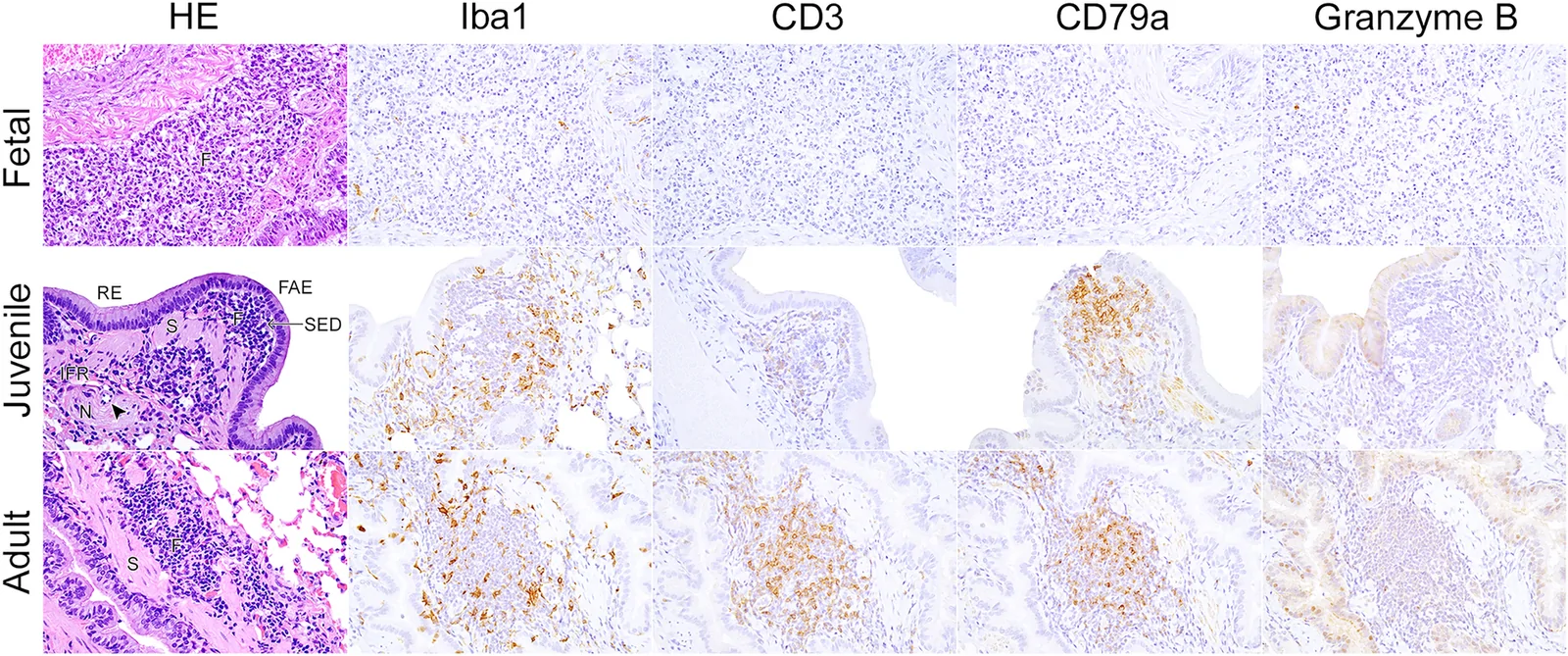
**Comparison of bronchus-associated lymphoid tissue (BALT) immunolabeling across fetal, juvenile, and adult Egyptian rousette (*Rousettus aegyptiacus*) tissues**. Sections are adjacent to major bronchioles; 40× magnification. Presumptive fetal BALT (areas of hypercellularity between large bronchioles and pulmonary vessels) has no immunoreactivity with CD3 and CD79a, and strong, rare positive immunoreactivity with Iba1 and Granzyme B. Juvenile and adult BALT have no-to-scant positive immunoreactivity with Granzyme B, but strong positive immunoreactivity with Iba1, CD3, and CD79a. Juvenile BALT displays the concentration of CD79a + cells at the apical border of the BALT follicle. Adult BALT did not have well-defined BALT follicles, likely from its relatively antigen-free managed care environment. FAE, follicle-associated epithelium; RE, respiratory epithelium; F, follicle; SED, subepithelial dome; IFR, interfollicular region; S, smooth muscle; N, nerve; arrowhead, high endothelial venule.

Throughout the image panels presented in this study, efforts were taken to include identical tissue sections across different immunohistochemical stains whenever technically feasible, thereby facilitating direct comparative analysis of staining patterns and cellular distributions. Comprehensive comparisons of Iba1, CD3, CD79a, and Granzyme B immunolabeling patterns across fetal, juvenile, and adult ERB bone marrow (Figure 11), thymus (Figure 12), lymph node (Figure 13), spleen (Figure 14), GALT (Figure 15), and BALT (Figure 16) are presented, providing detailed documentation of age-related changes in immune cell populations and their spatial distribution within lymphoid tissue architecture.

### Clinical pathology

Characterization of immune cell populations has been completed across diverse chiropteran species, including the Brazilian free-tailed bat (*Tadarida brasilensis*), Indian flying fox (*Pteropus giganteus*), and black flying fox (*Pteropus alecto*), among numerous other taxonomically distinct species [22–24, 66, 67]. Comprehensive hematological and clinical chemistry parameters for ERBs have been systematically compiled and analyzed by Rissmann et al. [68], providing essential baseline data for this epidemiologically significant species. However, considerable variation exists within reported parameter ranges, likely attributable to differences in analytical methodologies, reference panels, and automated analyzer systems employed across different research facilities [68–74]. Despite this inherent methodological variation, there exists consistent agreement across multiple studies that neither sex- nor age-related differences have been definitively identified in ERB hematological parameters [68, 71, 75].

ERB hematological analysis reveals distinctive morphological characteristics that differentiate this species from other mammalian taxa. Erythrocytes demonstrate mild polychromasia, reflecting the presence of young red blood cell populations within circulation [71, 76]. Neutrophils exhibit characteristic fine eosinophilic granulation within their cytoplasm, while lymphocytes and monocytes display typical morphological features with occasional nuclear indentation patterns. Eosinophils demonstrate distinctive morphological characteristics, featuring homogeneous, round, light-lavender-colored, small cytoplasmic granules that facilitate their identification within peripheral blood smears [71, 76].

Comparative analysis reveals that ERBs demonstrate several distinctive hematological characteristics relative to other chiropteran species. Specifically, ERBs exhibit slightly reduced hematocrit values, hemoglobin concentrations, total leukocyte counts, and lymphocyte counts compared with other bat species, while simultaneously demonstrating lower triglyceride levels [71]. Conversely, creatine kinase activity was elevated in ERBs relative to comparative chiropteran species. This finding may be reflective of common causes of CK elevation, including restraint, muscular injection, flying activity, microtraumas, or drug effects, or may potentially reflect species-specific metabolic adaptations or physiological requirements [71].

Serum protein electrophoretic analysis of ERB samples revealed a characteristic five-fraction pattern consisting of albumin, α-globulins, β1-globulins, β2-globulins, and γ-globulins [71]. This electrophoretic profile provides important baseline information regarding protein distribution patterns and can serve as a reference standard for detecting pathological alterations in protein metabolism or immune system function.

Manual differential leukocyte counts performed on 200 leukocytes from the 2 adult ERB specimens included in the present investigation are systematically presented in Table 2. These values demonstrate slightly elevated counts compared with those documented in current published literature, a variation that may reflect the advanced age of the specimens examined in this study [71]. Age-related alterations in leukocyte populations represent normal physiological adaptations that can influence differential count distributions and should be considered when interpreting hematological data from aged specimens. Due to morphological artifacts induced by prolonged formalin fixation, leukocyte morphological images are not provided within this manuscript. However, comprehensive photomicrographic documentation of ERB leukocyte morphology and morphological descriptions can be accessed in the published work of Moretti et al. [71], which provides an excellent reference resource for investigators requiring detailed cellular morphological information for this species.

**Table 2:** **Manual differential leukocyte counts of adult Egyptian rousettes (*Rousettus aegyptiacus*)**

	Neutrophils	Lymphocytes	Monocytes	Eosinophils	Basophils
Female (12 years old)	22.4	155.4	12.4	8.2	1.6
	11.2%	77.7%	6.2%	4.1%	0.8%
Male (10 years old)	18.5	160.75	11.25	6.75	2.75
	9.25%	80.375%	5.625%	3.375%	1.375%

Results per bat as presented as average count/200 cells (first line) and as the percentage of each cell type identified (second line). The average of four (male) or five (female) blood smear slides are presented herein.

## Discussion

This investigation represents the most comprehensive gross, histological, and immunohistological characterization of the lymphoid system in the Egyptian rousette (*Rousettus aegyptiacus*) documented to date, establishing a critical foundational resource for comparative pathology and immunological investigations in this epidemiologically significant reservoir species. Through systematic characterization of primary and secondary lymphoid organs across developmental stages, coupled with validation of cross-reactive immunohistochemical markers for key immune cell subsets, this work substantially advances our understanding of chiropteran immune architecture and provides essential interpretive context for experimental infection studies, immunological response assessments, and host–pathogen interaction dynamics in ERB populations.

The histologic atlas developed herein provides an in-depth reference for ERB lymphoid tissues across developmental stages, particular emphasis on juvenile specimens representing the established experimental model age group. Characterization of structural organization and cellular composition within primary and secondary lymphoid tissues, alongside systematic validation of immunohistochemical markers for key immune cell populations, reveals that ERB lymphoid organs demonstrate remarkable architectural conservation relative to other mammalian species, while simultaneously exhibiting nuanced variations that likely reflect functional adaptations to their unique ecological and physiological requirements. Notable species-specific architectural features include the presence of specialized periarterial macrophage sheaths and marginal zone bridging channels within splenic architecture, as well as the pronounced development of gut-associated lymphoid tissue (GALT) throughout the intestinal tract. These distinctive anatomical specializations may reflect evolutionary adaptations that facilitate enhanced immune surveillance and potentially contribute to the species’ capacity for viral disease tolerance and reservoir competence; however, the limited sample size and shared origin of the animals studied warrant cautious interpretation of this hypothesis.

Furthermore, the characterization of age- and tissue-specific immune cell distributions, encompassing CD3 + , CD79a + , Iba1 + , and Granzyme B + cell populations, provides compelling evidence supporting the hypothesis that immune tolerance mechanisms in chiropteran species are both developmentally modulated and spatially compartmentalized within lymphoid tissues. Comparative analysis with other chiropteran species reveals that ERBs retain many canonical lymphoid architectural features, yet demonstrate subtle but potentially significant differences in tissue organization and immune cell localization patterns. These architectural variations may represent key determinants underlying the species’ unique epidemiological role as a natural filovirus reservoir, warranting further investigation to elucidate their functional significance in host–pathogen interactions.

This investigation was subject to several inherent limitations that should be acknowledged when interpreting the findings. The relatively small sample size constrains statistical interpretation capabilities and limits the generalizability of results to broader ERB populations. All fetal tissue specimens were collected more than a decade prior to histological analysis and were subjected to extended formalin fixation periods, factors that likely compromised antigenicity and negatively impacted immunohistochemical staining quality and sensitivity. Certain MALT components, particularly palatine tonsils, were not identified histologically and therefore remain incompletely represented within this atlas. Additionally, while immunohistochemical analysis enabled successful identification of major lymphoid cell lineages, challenges associated with antibody cross-reactivity and marker sensitivity in non-model species precluded comprehensive characterization of all immune cell subtypes of potential interest. Lastly, this investigation was limited to morphological and basic immunohistochemical characterization and did not incorporate functional immunological assays, single-cell transcriptomic analysis, spatial transcriptomics, or flow cytometric analysis. The integration of these advanced methodological approaches would provide substantially enhanced insights into immune cell activation states, cellular diversity, and developmental ontogeny patterns.

Several promising avenues for future investigation emerge from the foundational work presented in this study. Expansion of histological and immunophenotypic studies to include larger specimen cohorts and wild-caught animals would facilitate comprehensive investigation of natural biological variability, sex- and age-based immunological differences, and environmental influences on immune architecture. Such expanded studies would also enable comparative analysis of lymphoid architecture in animals with documented coinfections involving multiple pathogenic agents. Additionally, the application of single-cell and spatial transcriptomic technologies would provide high-resolution insights into cell–cell interaction networks, tissue microenvironmental characteristics, and age-related immunological shifts within lymphoid tissues. These advanced analytical approaches would significantly enhance our understanding of the molecular mechanisms underlying immune tolerance and reservoir competence in this species. Further, functional characterization of mucosal immunity and MALT components, with particular emphasis on tonsillar and intestinal immune systems, represents a critical research priority that could substantially improve our understanding of initial pathogen encounter dynamics and viral shedding mechanisms. Such investigations would provide essential insights into the early stages of host–pathogen interactions and their role in maintaining persistent infections. Finally, comparative immunological studies across diverse chiropteran species, or between bats and other established reservoir host species, will continue to elucidate which immunological traits represent species-specific adaptations, pan-chiropteran characteristics, or convergent evolutionary strategies for viral disease tolerance.

This comprehensive investigation provides both a conceptual framework and practical resource for advancing our understanding of Egyptian rousette immunobiology, while simultaneously reinforcing the species’ value as a comparative model for emerging infectious disease research. The detailed anatomical and immunological characterization presented herein enhances our interpretive capacity for understanding infectious disease dynamics in ERB populations, while also providing findings with direct translational relevance for zoonotic disease modeling, vaccine development strategies, and public health preparedness initiatives in the context of emerging infectious disease threats. The establishment of this comprehensive lymphoid tissue atlas represents a critical step toward developing more sophisticated experimental models for investigating host–pathogen interactions, immune response mechanisms, and therapeutic intervention strategies. By providing detailed baseline characterization of normal lymphoid architecture and immune cell distributions, this work enables more precise interpretation of experimental perturbations and pathological alterations, ultimately advancing our capacity to understand and mitigate zoonotic disease risks at the human–wildlife interface.

## Data Availability

No datasets were generated or analyzed during the current study.
